# Cancer Incidence and Childhood Residence Near the Coldwater Creek Radioactive Waste Site

**DOI:** 10.1001/jamanetworkopen.2025.21926

**Published:** 2025-07-16

**Authors:** Michael Leung, Ian W. Tang, Joyce J. Y. Lin, Lorelei Mucci, Justin G. Farmer, Kaleigh McAlaine, Joseph J. Mangano, Marc G. Weisskopf

**Affiliations:** 1 Department of Environmental Health, Harvard T. H. Chan School of Public Health, Boston, Massachusetts; 2 Department of Epidemiology, Harvard T. H. Chan School of Public Health, Boston, Massachusetts; 3 Radiation and Public Health Project, Ocean City, New Jersey

## Abstract

**Importance:**

Beginning in the 1940s, radioactive waste from the effort to develop an atomic bomb was stored in the open near the St Louis, Missouri, airport, and over several decades contaminated nearby Coldwater Creek.

**Objective:**

To evaluate whether living near Coldwater Creek in childhood was associated with self-reported cancer incidence in the St Louis Baby Tooth–Later Life Health Study (SLBT).

**Design, Setting, and Participants:**

The SLBT cohort study was centered in St Louis and included individuals who donated their baby teeth from 1958 to 1970 to assess exposure to atmospheric nuclear weapons testing. Participants for the present analysis were recruited from June 22, 2021, to September 18, 2024.

**Exposures:**

Residential proximity to Coldwater Creek (≤1, >1 to 5, >5 to 20, and >20 km) in childhood when participants donated their baby teeth.

**Main Outcomes and Measures:**

Self-reported cancers, from which several composite outcomes were constructed: any cancer, solid cancers (all except leukemias and lymphomas), known radiosensitive cancers (thyroid, breast, leukemia, and basal cell), and nonradiosensitive cancers (all except thyroid, breast, leukemia, and basal cell). Odds ratios (OR) and 95% CIs between living near Coldwater Creek and several self-reported cancer outcomes were estimated, adjusting for sociodemographic characteristics and neighborhood median income.

**Results:**

There were 4209 eligible SLBT participants, of whom 2369 (56.3%) were female. The mean age at enrollment was 63 (range, 55-77) years. The background risk of any form of cancer was 24%. The OR was 1.44 (95% CI, 0.96-2.14) for any form of cancer comparing participants living 1 km or nearer with those living further than 20 km. There was a significant association for radiosensitive cancers (OR, 1.85; 95% CI, 1.21-2.81) but not for nonradiosensitive cancers (OR, 1.41; 95% CI, 0.86-2.30) for participants living 1 km or nearer vs further than 20 km. There was evidence of a dose-response association, where cancer risks were still elevated (albeit smaller) for those living in buffers of greater than 1 to 5 km and greater than 5 to 20 km. Despite imprecision in some of the site-specific estimates, clear signals for some organs, such as the thyroid, were still detected (OR for living ≤1 vs >20 km, 5.00; 95% CI, 1.23-20.32).

**Conclusions and Relevance:**

In this cohort study of baby teeth donors from the Greater St Louis area, evidence suggested a positive association between living near Coldwater Creek in childhood and risk of cancer.

## Key Points

**Question:**

Is there an association between cancer incidence and living near Coldwater Creek as a child when it was actively being contaminated by radioactive byproducts from the Manhattan Project?

**Findings:**

In this cohort study of 4209 participants, living near Coldwater Creek as a child was associated with an increased risk of overall cancer during long-term follow-up, with evidence of a dose-response association. When comparing those living around the creek or its floodplain (≤1 km) with those living further than 20 km away, the association with cancers known to be radiosensitive during childhood was stronger than that of nonradiosensitive cancers.

**Meaning:**

These findings suggest that childhood residential proximity to Coldwater Creek is associated with an increased risk of cancer, likely through radiation exposure associated with the creek.

## Teaser

This cohort study examines the association between living near Coldwater Creek, Missouri, as a child and self-report of cancer incidence collected via surveys administered in the St Louis Baby Tooth–Later Life Health Study.

## Introduction

**Figure 1.**
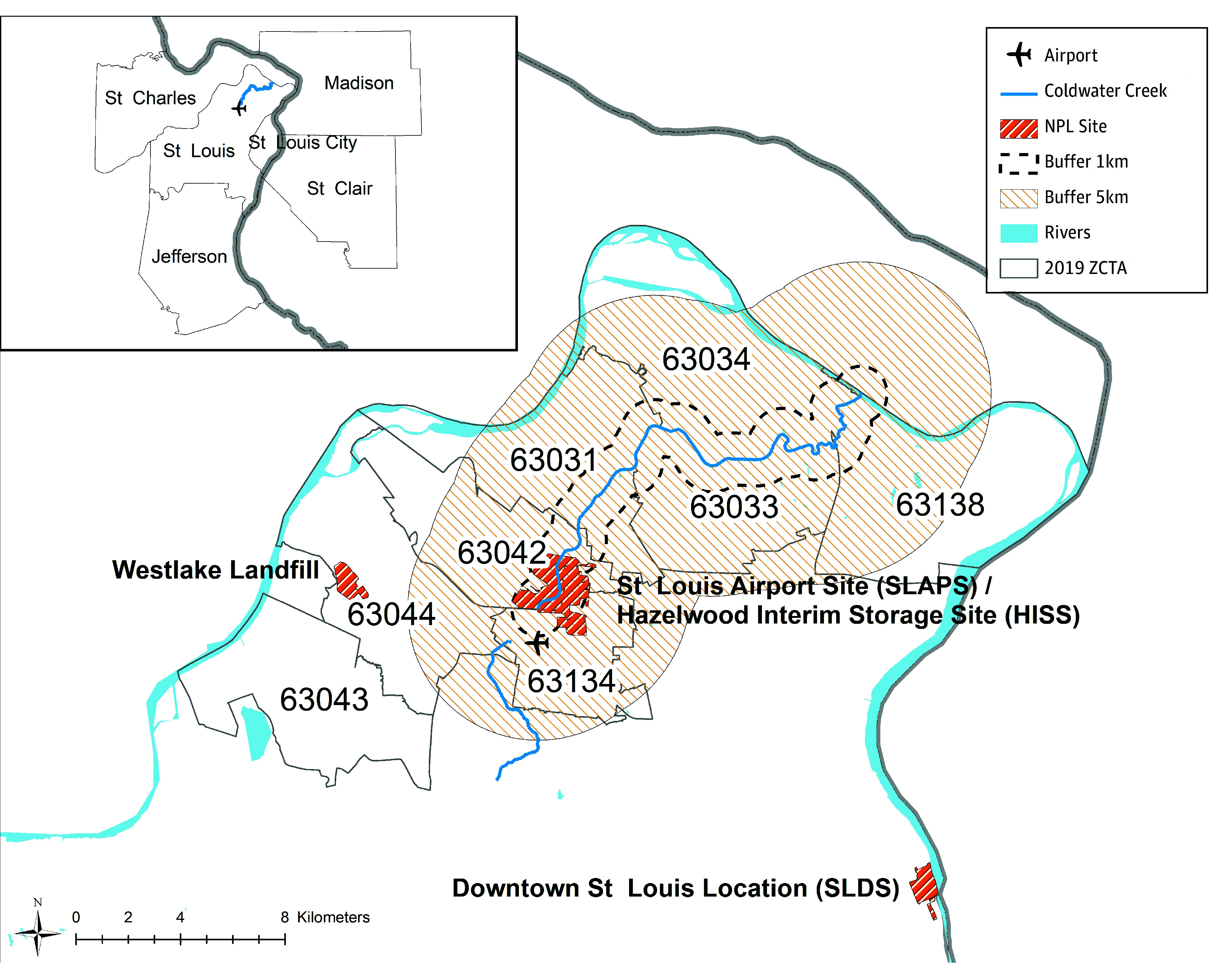
Buffers for Coldwater Creek and National Priority List (NPL) Sites Where Uranium Was Stored Data were obtained from 1960 census tracts in Missouri and Illinois; Coldwater Creek is a tributary of the Missouri River. Radioactive waste was stored at the St Louis Airport Site (SLAPS) in 1946 to 1966, and then subsequently, at the Hazelwood Interim Storage Site (HISS) in 1966 (SLAPS and HISS are within the same boundary). The portion of creek downstream (eastward) of SLAPS and HISS is considered radiologically contaminated from the storage of radioactive waste. The 2010 zip code tabulation area (ZCTA) used in the Missouri Department of Health and Senior Services analysis of cancer incidence rates are also shown.

St Louis, Missouri, played an important role in the development of the first atomic bombs.^1,2^ Under the Manhattan Project, Mallinckrodt Chemical Works processed and refined uranium in St Louis. Unlike some other important Manhattan Project locations in remote locations (eg, Los Alamos, New Mexico), Mallinckrodt Chemical Works was situated in the downtown of a major US city (Figure 1). With nowhere to store the radioactive waste at the downtown location, it was moved to a then rural area north of St Louis (Figure 1). Radioactive material at these sites was stored in drums or left uncovered on the ground, exposed to the elements, and so radiological contaminants leached into the nearby Coldwater Creek. Although it was only in the late 1980s that the US government officially acknowledged that Coldwater Creek was contaminated and began remedial activities, downstream communities potentially had been exposed to ionizing radiation for decades through both recreational (eg, playing in the creek) and residential (eg, breathing in dust suspended from floodplain soil when gardening) activities.^2^

The contamination of Coldwater Creek has raised concerns about an excess cancer risk among nearby residents,^1^ which prompted the Missouri Department of Health and Senior Services (MDHSS)^3^ and the Agency for Toxic Substances and Disease Registry (ATSDR)^2^ to evaluate the link between living near Coldwater Creek and developing cancer. Despite concluding that the overall cancer risk was low (ATSDR)^2^ and that the pattern of cancers was not consistent with what would be expected from radiation exposure (MDHSS),^3^ these findings need to be interpreted with caution, as both assessments have some important limitations. The MDHSS study reflected a contemporary examination of cancer occurrence among those living near Coldwater Creek from 1996 to 2011 after remediation efforts had started (ie, many would not have lived there as children before remediation). Furthermore, the ATSDR study, although it relied on precise intake estimates based on environmental sampling data from the mid-1980s onward, had a lot of room for error in reconstructing possible past radiological intake and lifetime cancer risks for individuals who hypothetically would have lived near the creek decades prior.

In this study, information derived from the St Louis Baby Tooth–Later Life Health Study (SLBT),^4,5^ a cohort centered in St Louis, was used to provide an analysis that is complementary to the MDHSS and ATSDR assessments. Although we do not have precise radiation contamination exposure estimates, we have childhood addresses for all our participants in the 1940s to 1960s and so could examine the association between living near Coldwater Creek as a child before remediation and self-report of cancer incidence collected via surveys administered at SLBT enrollment.

## Methods

### Study Population

The SLBT has been described previously.^4^ Briefly, our study population is a subsample of the St Louis Baby Tooth Survey started in 1958,^5^ for which individuals donated their baby teeth to assess exposure to atmospheric nuclear testing (thus, participants had no recognition when donating that the creek would be a health concern). From June 22, 2021, to September 18, 2024, we recruited 5361 of the approximately 55 000 original tooth donors born between 1945 and 1966 (eFigure 1 in Supplement 1). Of these, we excluded those who had childhood residential addresses that could not be geocoded. We also restricted our study population to those who lived within the Greater St Louis area in childhood (eTable 1 in Supplement 1). Participants provided written informed consent prior to participating in SLBT. We followed the Strengthening the Report of Observational Studies in Epidemiology (STROBE) guideline for cohort studies. The institutional review board of Harvard T. H. Chan School of Public Health approved this study.

### Proximity to Coldwater Creek

For each participant, we calculated the shortest Euclidean distance (in kilometers) between Coldwater Creek and their reported childhood address when they donated their teeth. We also considered 4 categorical exposure levels based on where participants lived: 1 km or nearer, further than 1 to 5 km, further than 5 to 20 km, and further than 20 km from Coldwater Creek (Figure 1). We used the 1-km buffer to capture potentially high radiation exposure not only from living near the creek but also the increased likelihood of playing in the creek because of residential proximity. We used the 5-km cutoff, as this was approximately the size of the study area in the MDHSS analysis.^3^ Finally, we used cutoff greater than 20 km as our reference population as these individuals were unlikely to visit the creek given the distance and so would not have been exposed to creek-related radiation. We used residential proximity as a proxy for creek-related radiation exposure, since we currently do not have radionuclide measurements from the participants’ teeth.

### Cancer Outcomes and Covariates

Health and sociodemographic data were collected through self-report from surveys administered at enrollment. Self-reported cancer diagnoses were grouped into 4 composite cancer outcomes: (1) all cancers, which is a broad outcome category of public health importance often examined in radiation epidemiology,^6^ and justified here because broad outcome categories based on self-report diagnoses may have better specificity than narrow categories of disease; (2) solid cancers, often examined in radiation studies that separate leukemias and lymphomas from solid cancer diagnoses^6^; (3) known radiosensitive cancers during childhood, which include breast, thyroid, leukemia, and basal cell skin cancer^6,7^; and (4) nonradiosensitive cancers, which we would expect to have a weaker association than that for radiosensitive cancers. The list of cancers within each category and their ascertainment are shown in eTable 2 in Supplement 1.

Information on relevant covariates was also obtained through self-report. We included in our models sex, race and ethnicity, father’s educational attainment, economic status at 12 years of age, and birth decades. Race and ethnicity were categorized as American Indian or Alaska Native, Asian, Black, Hispanic White, and those who preferred not to say, collectively termed *Other*, or non-Hispanic White. Race and ethnicity were included because many social factors contributed to where people of different races lived and thus their proximity to the creek and influence risk of cancer by race.^8,9^ The original self-reported categories of these variables and how they were operationalized are reported in eTable 3 in Supplement 1. We also considered neighborhood-level median income, which we obtained from the 1960 decennial census.^10^

### Statistical Analysis

For each cancer outcome, we fitted a generalized additive model^11^ to examine the association between distance from the creek and the incidence of cancer, adjusting for the covariates described above. Our generalized additive models showed that although the association was approximately linear for the cancer outcomes, there was high variance in the estimate past approximately 20 km (eFigures 2-5 in Supplement 1). Thus, we chose instead to present as our primary analysis the odds ratio (OR) for the association with living 1 km or nearer, further than 1 to 5 km, and further than 5 to 20 km from Coldwater Creek compared with those further than 20 km estimated from a logistic regression. We also tested for a linear trend in the estimates using α = .05 by converting the exposure categories as an ordered factor coded with a linear contrast.

We used the parametric g-formula^12^ to estimate (1) the background counterfactual risk of cancer (had they lived >20 km) and (2) the additional number of cases per 10 000 individuals, such that we could compare our estimates with those of the ATSDR report.^2^ Furthermore, we stratified all analyses by biological sex, as previous literature suggested that there may be differential radiosensitivity by sex.^6^

We also conducted multiple sensitivity analyses. First, we used multiple imputation by chained equations to account for any missingness in the covariates.^13,14^ Second, we explored whether the findings would change if we treated the missing cancer data as “no cancer” rather than missing at random, as presumably those with cancer would report their cancer. Third, we excluded participants with cancers diagnosed within the last 25 years, as childhood and adult cancers are etiologically different.^6^ Fourth, the incidence of prostate, thyroid, and skin cancers is highly sensitive to screening intensity and so we re-examined the association with overall cancer excluding these 3 cancers. Fifth, we considered an alternate definition of radiosensitive cancers based on findings by the US Nuclear Regulatory Commission.^15^ Sixth, we calculated E values to assess the magnitude of confounding that would be needed to explain away the point estimate.^16^ Finally, we examined the extent to which self-selection into SLBT could bias our findings, given that the radioactivity of Coldwater Creek and its potential link to cancer incidence has garnered substantial press. To do so, we looked at whether distance was associated with being in SLBT. All analyses were performed in R, version 4.1.0 (R Foundation for Statistical Computing),^17^ and Two-sided *P* < .05 indicated statistical significance.

## Results

There were 4209 eligible SLBT participants for our analysis (eFigure 1 in Supplement 1 and Table 1). The mean age at enrollment was 63 (range, 55-77) years; most participants were female (2369 [56.3%]); 1808 were male (43.0%) and 32 were missing sex data (0.8%). A total of 4012 participants (95.3%) were non-Hispanic White and 197 (4.7%) were categorized as Other. Participants 1 km or nearer were mostly non-Hispanic White (137 of 139 [98.6%]), had fathers with a more than high school education (83 of 139 [59.7%]), and considered their household economic status at 12 years of age to be middle income (115 of 139 [82.7%]). Most participants 1 km or nearer were born in the 1950s or 1960s and lived in a neighborhood with a mean (SD) income of approximately $7100 ($210). We found that the distributions of these characteristics were similar among participants who lived further than 1 to 5 km, further than 5 to 20 km, and further than 20 km, except that their neighborhood mean (SD) income was higher ($7500 [$880] for >1 to 5 km, $8200 [$2900] for >5 to 20 km, and $7200 [$1100] for >20 km).

**Table 1.** Characteristics of the Study Population by Proximity to Coldwater Creek, Missouri

Characteristic^a^	Childhood residential proximity to Coldwater Creek, km			
	≤1 (n = 139)	>1 to 5 (n = 609)	>5 to 20 (n = 2086)	>20 (n = 1375)
Sex, No. (%)				
Female	83 (59.7)	348 (57.1)	1168 (56.0)	770 (56.0)
Male	55 (39.6)	258 (42.4)	901 (43.2)	594 (43.2)
Missing	1 (0.7)	3 (0.5)	17 (0.8)	11 (0.8)
Race and ethnicity, No. (%)				
Non-Hispanic White	137 (98.6)	595 (97.7)	1954 (93.7)	1326 (96.4)
Other^b^	2 (1.4)	14 (2.3)	132 (6.3)	49 (3.6)
Father’s educational attainment, No. (%)				
High school or less	55 (39.6)	245 (40.2)	688 (33.0)	593 (43.1)
Associate’s degree or some college	30 (21.6)	113 (18.6)	346 (16.6)	269 (19.6)
College	33 (23.7)	151 (24.8)	474 (22.7)	269 (19.6)
More than college	20 (14.4)	91 (14.9)	533 (25.6)	221 (16.1)
Missing	1 (0.7)	9 (1.5)	45 (2.2)	23 (1.7)
Economic status at 12 y of age, No. (%)				
Low income	10 (7.2)	50 (8.2)	178 (8.5)	114 (8.3)
Middle income	115 (82.7)	502 (82.4)	1515 (72.6)	1107 (80.5)
High income	14 (10.1)	57 (9.4)	379 (18.2)	153 (11.1)
Missing	0	0	14 (0.7)	1 (0.1)
Birth decade, No. (%)				
1940s	1 (0.7)	5 (0.8)	27 (1.3)	9 (0.7)
1950s	74 (53.2)	351 (57.6)	1322 (63.4)	829 (60.3)
1960s	64 (46.0)	252 (41.4)	727 (34.9)	535 (38.9)
Missing	0	1 (0.2)	10 (0.5)	2 (0.1)
Median income from the 1960 census, mean (SD), US $^c^	7100 (210)	7500 (880)	8200 (2900)	7200 (1100)

^a^ Data were obtained from 1945 to 1966. Percentages have been rounded and may not total 100.

^b^ Includes Black, American Indian or Alaska Native, Asian, Black, Hispanic White, Native Hawaiian or Pacific Islander, and those who preferred not to say.

^c^ Each participant was assigned the median income of the census tract in which they resided.

There were 1009 individuals (24.0%) who reported having cancer, with a higher proportion for those living closer to Coldwater Creek (39 of 130 [30.0%]≤1 km; 164 of 583 [28.1%]>1 to 5 km; 495 of 1956 [25.3%]>5 to 20 km; and 311 of 1303 [23.9%]>20 km) (Table 2). This pattern also generally applied to site-specific cancers (eTable 4 in Supplement 1). Our estimated background counterfactual risks for the different cancers (ie, adjusted for the covariates) are presented in eTable 5 in Supplement 1. Overall, we would expect 24% of our participants to be diagnosed with any form of cancer had they lived further than 20 km from the site (similar to Table 1).

**Table 2.** Cancer Status Through 55 to 77 Years of Age Stratified by Proximity to Coldwater Creek, Missouri

Cancer type	Residential proximity to Coldwater Creek, No. (%)^a^			
	≤1 km (n = 139)	>1-5 km (n = 609)	>5-20 km (n = 2086)	>20 km (n = 1375)
**Any^b^**				
Noncase	91 (70.0)	419 (71.9)	1461 (74.7)	992 (76.1)
Case	39 (30.0)	164 (28.1)	495 (25.3)	311 (23.9)
Missing	9	26	130	72
**Solid^c^**				
Noncase	91 (70.0)	421 (72.1)	1477 (75.0)	1006 (77.1)
Case	39 (30.0)	163 (27.9)	492 (25.0)	298 (22.9)
Missing	9	25	117	71
**Radiosensitive^d^**				
Noncase	104 (75.4)	490 (81.8)	1680 (83.3)	1136 (84.6)
Case	34 (24.6)	109 (18.2)	336 (16.7)	207 (15.4)
Missing	1	10	70	32
**Nonradiosensitive^e^**				
Noncase	108 (83.1)	494 (84.2)	1703 (86.0)	1132 (86.4)
Case	22 (16.9)	93 (15.8)	277 (14.0)	178 (13.6)
Missing	9	22	106	65

^a^ Number of missing were not included in the calculation of percentages. Data were obtained from 1945 to 1966.

^b^ Includes breast, endometrial, cervical, ovarian, uterine, prostate, testicular, lung, colon, bladder, kidney, leukemia, liver, thyroid, non-Hodgkin lymphoma, pancreas, melanoma, basal cell skin, squamous cell skin, and Hodgkin lymphoma.

^c^ Includes breast, endometrial, cervical, ovarian, uterine, prostate, testicular, lung, colon, bladder, kidney, liver, thyroid, pancreas, melanoma, basal cell skin, and squamous cell skin.

^d^ Includes breast, thyroid, leukemia, and basal cell skin.

^e^ Includes endometrial, cervical, ovarian, uterine, prostate, testicular, lung, colon, bladder, kidney, liver, non-Hodgkin lymphoma, pancreas, melanoma, squamous cell skin, and Hodgkin lymphoma.

**Figure 2.**
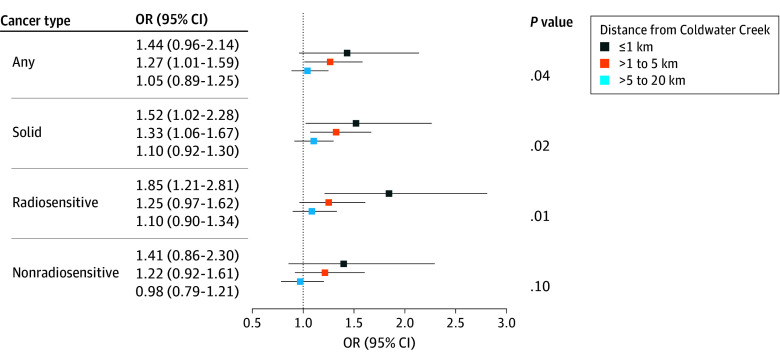
Estimates for the Association Between Composite Cancer Outcomes and Proximity to Coldwater Creek Cancer outcomes included any, solid, radiosensitive, and nonradiosensitive cancers. Proximity was defined as living 1 km or nearer, further than 1 to 5 km, and further than 5 to 20 km compared with living further than 20 km from Coldwater Creek, Missouri, in 1945 to 1966. Estimates were adjusted for sex, race and ethnicity, father’s educational attainment, economic status at 12 years of age, birth decade, and mean income from the 1960 decennial census. The points represent odds ratios (ORs), and the error bars represent 95% CI. *P* values were calculated from a trend test to assess whether the OR decreases linearly with distance. Additional data can be found in eTable 6 in Supplement 1.

Overall risk of cancer for residence 1 km or nearer was more elevated than for further distances, although not statistically significant (OR, 1.44; 95% CI, 0.96-2.14), while risk of solid cancers (OR, 1.52; 95% CI, 1.02-2.28) and risk of radiosensitive cancers (OR, 1.85; 95% CI, 1.21-2.81) were statistically elevated compared with those who resided farther than 20 km from the site (Figure 2 and eTable 6 in Supplement). Among those living further than 1 to 5 km, the overall risk of cancer was still elevated with an OR of 1.27 (95% CI, 1.01-1.59) for any cancer, 1.33 (95% CI, 1.06-1.67) for solid cancers, 1.25 (95% CI, 0.97-1.62) for radiosensitive cancers, and 1.22 (95% CI, 0.92-1.61) for nonradiosensitive cancers. Among those living further than 5 to 20 km, the ORs were above 1 (except for nonradiosensitive cancers), but none were statistically significant. There was also evidence of a linear dose-response for all cancer outcomes except for nonradiosensitive cancers, where *P* = .04 for trend for any cancer, *P* = .02 for solid cancers, *P* = .003 for radiosensitive cancers, and *P* = .10 for nonradiosensitive cancers (Figure 2).

Associations with site-specific cancers are shown in eTable 6 in Supplement 1, and corresponding additive estimates are presented in eTable 7 in Supplement 1. In our sex-stratified analyses, we found that the odds ratios among male participants were greater than those in female participants (eFigure 6 and eTable 8 in Supplement 1). For example, among male participants, the OR was 2.33 (95% CI, 1.27-4.29) for any cancer and 3.25 (95% CI, 1.70-6.20) for radiosensitive cancers; among female participants, the ORs were 1.02 (95% CI, 0.59-1.77) and 1.32 (95% CI, 0.76-2.31), respectively.

In our sensitivity analyses, we found that multiple imputation, treating the missing cancer data as no cancer, and excluding individuals with childhood cancers produced similar findings (eFigure 7 and eTables 9-11 in Supplement 1). When excluding screening-sensitive cancers, the risk was attenuated, though the 95% CIs were wider, especially for those 1 km or nearer because there were only 11 cases within this buffer (eFigure 7 and eTable 12 in Supplement 1). When using the alternate definition of radiosensitive, we found that the odds ratios of radiosensitive cancers were no longer larger than those of nonradiosensitive cancers (eFigure 7 and eTable 13 in Supplement 1). In our sensitivity analysis of potential unmeasured confounding, some E values were large (eg, E = 9.47 for thyroid), which suggests that unmeasured or residual confounding is unlikely to explain away the associations (eTable 14 in Supplement 1). Finally, in our assessment of potential self-selection, we found that individuals living 20 km or nearer were less likely to participate in the SLBT (eTable 15 in Supplement 1).

## Discussion

In this cohort study, we used data from the SLBT to gain insight into the potential carcinogenic effects of living near Coldwater Creek as a child when it was actively being contaminated by radioactive byproducts from the wartime processing of uranium.^1^ We found that participants living 1 km or nearer had a higher risk of cancer compared with those further than 20 km, which perhaps points to creek-related radiation exposure as the likely culprit. It is also important to highlight that the organs we found to be radiosensitive (eg, thyroid) were different from those identified by the US Nuclear Regulatory Commission.^15^ This is not surprising, given that their findings were based on populations exposed to acute whole-body external irradiation (eg, atomic bomb survivors), whereas our participants experienced chronic low-dose external and/or internal irradiation, which could lead to different pathways of radiation carcinogenesis.^6^

We found evidence for a dose-response where the risk of cancer among those living further than 1 to 5 km was elevated, but the association was attenuated for those further than 5 to 20 km. This may be because as far as 5 km, individuals may still travel to the creek for recreational activities. It might also suggest that radiation exposures probably extended beyond the creek’s floodplain, which can be partially attributed to the post–World War II housing boom in the 1950s to 1970s, which effectively redistributed the contamination throughout North St Louis County.^1^ It is also important to note for participants further than 1 km, the associations with what we considered radiosensitive cancers were no longer greater than those with nonradiosensitive cancers. This was mainly driven by the null associations with breast cancer and leukemia, despite the strong signal for thyroid cancer (and to a lesser extent basal cell skin cancer). In general, these findings point to the potential importance of assessing radiation exposures beyond the immediate floodplain.

We also found that the associations among male participants appeared greater than those in female participants living 1 km or nearer compared with their counterparts living further than 20 km. This is somewhat compatible with previous literature on differential radiosensitivity by biological sex; although many cancer studies show that female individuals are generally more radiosensitive due to certain biological differences (eg, hormonal influences),^6^ some work has shown that for some radiosensitive cancers such as leukemia, the risk in male individuals appeared higher for certain subtypes such as acute myeloid leukemia.^18^ Again, past evidence was mostly based on atomic bomb survivors, which may not be generalizable to our context; and so it is unclear whether our findings reflect true biological differences in sensitivity to chronic low-dose radiation, or whether the sex-stratified analyses reflect differences in behaviors (eg, boys may be more likely to spend more time at the creek) and/or interactions with other cancer risk factors (eg, smoking). In mouse models, radiation effects on leukemia are stronger in male than female mice, which appeared related to differential stem cell sensitivity and hematopoietic dynamics.^19^

Overall, we observed a pattern of cancer incidences that was different from that of the MDHSS analysis.^3^ Our findings more strongly suggest that the pattern is compatible with radiation-induced cancers, given that we found a consistently elevated risk with radiosensitive cancers, whereas the other investigators did not. The MDHSS investigators may not have seen a pattern more consistent with radiation-induced cancers because they only considered people who lived there at the time they did their study (and not as children before the 1990s), while our study focused on childhood exposures. This is an important distinction, as evidence shows that radiosensitivity is age-dependent for several cancers. For example, thyroid cancer is known to be most sensitive to radiation in childhood,^6,20^ while it appears to have very low radiosensitivity if exposure is in adulthood,^6^ which could explain the MDHSS findings.

It is also difficult to compare our findings with the ATSDR assessment^2^ because the ATSDR investigators did not link historical exposure to individual cancer cases. Instead, they attempted to reconstruct the radiological intake decades prior based on several different assumptions (eg, soil intake from creek-related activities). Our approach does not require such assumptions, as it does not define a specific level of radiation exposure. Although not all people living within our buffers would experience the same exposure, to the extent there is error in this assignment, it would likely bias associations toward the null. We found the additional risk of cancer associated with Coldwater Creek to be much larger than that reported in the ATSDR report (eTable 16 in Supplement 1); overall, we found that living 1 km or nearer to Coldwater Creek would yield about 700 more cases per 10 000 population (a 29% increase in the risk of any cancer); further than 1 to 5 km, about 450 more cases per 10 000 (a 19% increase in risk); and further than 5 to 20 km, about 90 more cases per 10 000 (a 4% increase in risk). These findings are more compatible with those of a similar contamination scenario in what is now Russia.^21,22,23^ Thus, the comparatively smaller associations reported in the ATSDR assessment are most likely due to a combination of (1) underestimating some of the inputs that generated their radiological intake estimates, which they note is possible given that the “waste entered the creek decades ago, and detailed information about how it moved with sediment and into the floodplain does not exist”^2^; (2) using the lifetime attributable risk coefficients for low doses of ionizing radiation (eg, medically irradiation treatment),^24^ but these may not be applicable here, as past residents were chronically exposed to very raw materials used in the production of the atomic bomb; and/or (3) potentially oversimplifying their methodology for estimating lifetime risks with the intention of improving accessibility to the general public.^2^ Finally, it is also important to note that their assessment ignores other contaminants that may have been present (eg, jet fuel, heavy metals)^2^ and the psychosocial impact of living near a cancer hotspot, in that residents living near contaminated areas may experience chronic stress,^25^ which could not only independently cause cancers^26^ but also perhaps magnify sensitivity to radiation exposure.^27^

### Strengths and Limitations

There are several notable strengths of our study. First, we had childhood addresses, which allowed us to examine the associations between childhood exposures to radiation from Coldwater Creek in the 1940s through 1960s and cancer incidence. Second, we calculated and presented the excess number of cases, but these should be interpreted with caution as they are contingent on the associations being causal. Although this cannot be verified using observational data, we did have rich information on several individual- and area-level confounders. Last, we showed in our sensitivity analyses that our findings were robust to several assumptions, and that potential biases (eg, unmeasured confounding) were unlikely to fully explain our associations.

Our study also had several limitations. First, our outcomes were self-reported, and so there may be errors in reporting site-specific cancers. However, our composite cancer outcomes should be relatively robust to this error, as participants should accurately report any vs no cancer. Second, we had a relatively small sample size to study associations with site-specific cancers, which led to some unstable estimates. This also meant that we were not able to examine effect modification or incorporate the age of diagnosis (eg, in a Cox proportional hazards model), as we could not stratify the data further. Moreover, there was potential selection bias, as we were conditioning on cancer survival for enrollment into our study—that is, those with cancer may have died or were less likely to participate in our later-life study (eFigure 8 in Supplement 1). If this were related to living near Coldwater Creek, which is possible given that we found that those living 20 km or nearer were less likely to participate, we would expect this to bias results toward the null. Finally, our results may not be generalizable to the entire Greater St Louis area, as non-Hispanic White individuals are overrepresented in our sample.

## Conclusions

In this cohort study, we used the SLBT to provide evidence that living near Coldwater Creek was associated with increased cancer risk. Our study was able to address issues that the MDHSS and ATSDR assessments could not, and so provides a complementary analysis of the potential effects of preremediation childhood exposures to radiological contaminants in Coldwater Creek. As we continue to recruit more participants into the SLBT and continue follow-up of existing participants, we may soon be able to improve the precision of our estimates, as well as explore factors that could drive effect heterogeneity.

## Notes

Supplement 1. **eFigure 1.** Flowchart of SLBT Participant Selection **eFigure 2.** Association Between Distance From Coldwater Creek and the Incidence of Any Form of Cancer **eFigure 3.** Association Between Distance From Coldwater Creek and the Incidence of Solid Cancer **eFigure 4.** Association Between Distance From Coldwater Creek and the Incidence of Radiosensitive Cancer **eFigure 5.** Association Between Distance From Coldwater Creek and the Incidence of Nonradiosensitive Cancer **eFigure 6.** Sex-Stratified Estimates for the Association Between Composite Cancer Outcomes and Proximity to Coldwater Creek **eFigure 7.** Sensitivity Analyses Estimates for the Association Between Composite Cancer Outcomes and Proximity to Coldwater Creek **eFigure 8.** A Directed Acyclic Graph Representing the Bias That Could Arise From Conditioning on Cancer Survival and Study Participation **eTable 1.** List of Counties in Greater St. Louis **eTable 2.** List of Cancers for Each Composite Outcome **eTable 3.** Operationalization of Analytic Variables **eTable 4.** Site-Specific Cancer Status Through Ages 55 to 77 Stratified by Proximity to Coldwater Creek, Missouri, 1945 to 1966 **eTable 5.** Background Counterfactual Risks Obtained Through G-Computation for Several Cancer Outcomes **eTable 6.** Estimates for the association between several cancer outcomes and Proximity to Coldwater Creek, Missouri, 1945 to 1966 **eTable 7.** Additional Cases per 10 000 for the Association Between Several Cancer Outcomes and Proximity to Coldwater Creek, Missouri, 1945 to 1966 **eTable 8.** Sex-Stratified Estimates for the Association Between Several Cancer Outcomes and Proximity to Coldwater Creek, Missouri, 1945 to 1966 **eTable 9.** Estimates for the Association Between Several Cancer Outcomes and Proximity to Coldwater Creek, Missouri, 1945 to 1966, After Multiple Imputation **eTable 10.** Estimates for the Association Between Several Cancer Outcomes and Proximity to Coldwater Creek, Missouri, 1945 to 1966, After Treating Missing Cancer Outcomes as No Cancer **eTable 11.** Estimates for the Association Between Several Cancer Outcomes and Proximity to Coldwater Creek, Missouri, 1945 to 1966, After Excluding Individuals With Childhood Cancers **eTable 12.** Estimates for the Association Between Any Cancer and Proximity to Coldwater Creek, Missouri, 1945 to 1966, After Restricting to Cancers That Are Not Sensitive to Screening Intensity **eTable 13.** Estimates for the Association Between Radiosensitive Cancer and Proximity to Coldwater Creek, Missouri, 1945 to 1966, After Using an Alternate Definition of “Radiosensitive” Based on Cancers Identified by the US Nuclear Regulatory Commission **eTable 14.** E Values for the Estimates From the Primary Analysis to Assess for the Magnitude of Confounding That Would Be Needed to Explain Away the Point Estimate **eTable 15.** Estimates for the Association Between SLBT Participation and Proximity to Coldwater Creek, Missouri, 1945 to 1966 **eTable 16.** Comparison of Our Additive Estimates (Excess Cases per 10 000) from the St. Louis Baby Tooth–Later Life Health Study (SLBT) With Those From the Agency for Toxic Substances and Disease Registry (ATSDR) Report

Supplement 2.
Data Sharing Statement
