# Supplementary material for: Cancer Incidence and Childhood Residence Near the Coldwater Creek Radioactive Waste Site
Source: JAMA Netw Open. 2025 Jul 16;8(7):e2521926. doi: 10.1001/jamanetworkopen.2025.21926 (PMC12268482; doi:10.1001/jamanetworkopen.2025.21926)
Supplement: Supplement 1. — eFigure 1. Flowchart of SLBT Participant Selection eFigure 2. Association Between Distance From Coldwater Creek and the Incidence of Any Form of Cancer eFigure 3. Association Between Distance From Coldwater Creek and the Incidence of Solid Cancer eFigure 4. Association Between Distance From Coldwater Creek and the Incidence of Radiosensitive Cancer eFigure 5. Association Between Distance From Coldwater Creek and the Incidence of Nonradiosensitive Cancer eFigure 6. Sex-Stratified Estimates for the Association Between Composite Cancer Outcomes and Proximity to Coldwater Creek eFigure 7. Sensitivity Analyses Estimates for the Association Between Composite Cancer Outcomes and Proximity to Coldwater Creek eFigure 8. A Directed Acyclic Graph Representing the Bias That Could Arise From Conditioning on Cancer Survival and Study Participation eTable 1. List of Counties in Greater St. Louis eTable 2. List of Cancers for Each Composite Outcome eTable 3. Operationalization of Analytic Variables eTable 4. Site-Specific Cancer Status Through Ages 55 to 77 Stratified by Proximity to Coldwater Creek, Missouri, 1945 to 1966 eTable 5. Background Counterfactual Risks Obtained Through G-Computation for Several Cancer Outcomes eTable 6. Estimates for the association between several cancer outcomes and Proximity to Coldwater Creek, Missouri, 1945 to 1966 eTable 7. Additional Cases per 10 000 for the Association Between Several Cancer Outcomes and Proximity to Coldwater Creek, Missouri, 1945 to 1966 eTable 8. Sex-Stratified Estimates for the Association Between Several Cancer Outcomes and Proximity to Coldwater Creek, Missouri, 1945 to 1966 eTable 9. Estimates for the Association Between Several Cancer Outcomes and Proximity to Coldwater Creek, Missouri, 1945 to 1966, After Multiple Imputation eTable 10. Estimates for the Association Between Several Cancer Outcomes and Proximity to Coldwater Creek, Missouri, 1945 to 1966, After Treating Missing Cancer Outcomes as No Cancer eTable 11. Esti [file jamanetwopen-e2521926-s001.pdf]

# Supplementary Online Content

Leung M, Tang IW, Lin JJY, et al. Cancer incidence and childhood residence near the Coldwater Creek radioactive waste site. *JAMA Netw Open.* 2025;8(7):e2521926. doi:10.1001/jamanetworkopen.2025.21926

**eFigure 1.** Flowchart of SLBT Participant Selection

**eFigure 2.** Association Between Distance From Coldwater Creek and the Incidence of Any Form of Cancer

**eFigure 3.** Association Between Distance From Coldwater Creek and the Incidence of Solid Cancer

**eFigure 4.** Association Between Distance From Coldwater Creek and the Incidence of Radiosensitive Cancer

**eFigure 5.** Association Between Distance From Coldwater Creek and the Incidence of Nonradiosensitive Cancer

**eFigure 6.** Sex-Stratified Estimates for the Association Between Composite Cancer Outcomes and Proximity to Coldwater Creek

**eFigure 7.** Sensitivity Analyses Estimates for the Association Between Composite Cancer Outcomes and Proximity to Coldwater Creek

**eFigure 8.** A Directed Acyclic Graph Representing the Bias That Could Arise From Conditioning on Cancer Survival and Study Participation

**eTable 1.** List of Counties in Greater St. Louis

**eTable 2.** List of Cancers for Each Composite Outcome

**eTable 3.** Operationalization of Analytic Variables

**eTable 4.** Site-Specific Cancer Status Through Ages 55 to 77 Stratified by Proximity to Coldwater Creek, Missouri, 1945 to 1966

**eTable 5.** Background Counterfactual Risks Obtained Through G-Computation for Several Cancer Outcomes

**eTable 6.** Estimates for the association between several cancer outcomes and Proximity to Coldwater Creek, Missouri, 1945 to 1966

**eTable 7.** Additional Cases per 10 000 for the Association Between Several Cancer Outcomes and Proximity to Coldwater Creek, Missouri, 1945 to 1966

**eTable 8.** Sex-Stratified Estimates for the Association Between Several Cancer Outcomes and Proximity to Coldwater Creek, Missouri, 1945 to 1966

**eTable 9.** Estimates for the Association Between Several Cancer Outcomes and Proximity to Coldwater Creek, Missouri, 1945 to 1966, After Multiple Imputation

**eTable 10.** Estimates for the Association Between Several Cancer Outcomes and Proximity to Coldwater Creek, Missouri, 1945 to 1966, After Treating Missing Cancer Outcomes as No Cancer

**eTable 11.** Estimates for the Association Between Several Cancer Outcomes and Proximity to Coldwater Creek, Missouri, 1945 to 1966, After Excluding Individuals With Childhood Cancers

**eTable 12.** Estimates for the Association Between Any Cancer and Proximity to Coldwater Creek, Missouri, 1945 to 1966, After Restricting to Cancers That Are Not Sensitive to Screening Intensity

© 2025 Leung M et al. *JAMA Network Open*.

**eTable 13.** Estimates for the Association Between Radiosensitive Cancer and Proximity to Coldwater Creek, Missouri, 1945 to 1966, After Using an Alternate Definition of "Radiosensitive" Based on Cancers Identified by the US Nuclear Regulatory Commission
**eTable 14.** E Values for the Estimates From the Primary Analysis to Assess for the Magnitude of Confounding That Would Be Needed to Explain Away the Point Estimate
**eTable 15.** Estimates for the Association Between SLBT Participation and Proximity to Coldwater Creek, Missouri, 1945 to 1966
**eTable 16**. Comparison of Our Additive Estimates (Excess Cases per 10 000) from the St. Louis Baby Tooth–Later Life Health Study (SLBT) With Those From the Agency for Toxic Substances and Disease Registry (ATSDR) Report

This supplementary material has been provided by the authors to give readers additional information about their work.

**eFigure 1.** Flowchart of SLBT Participant Selection

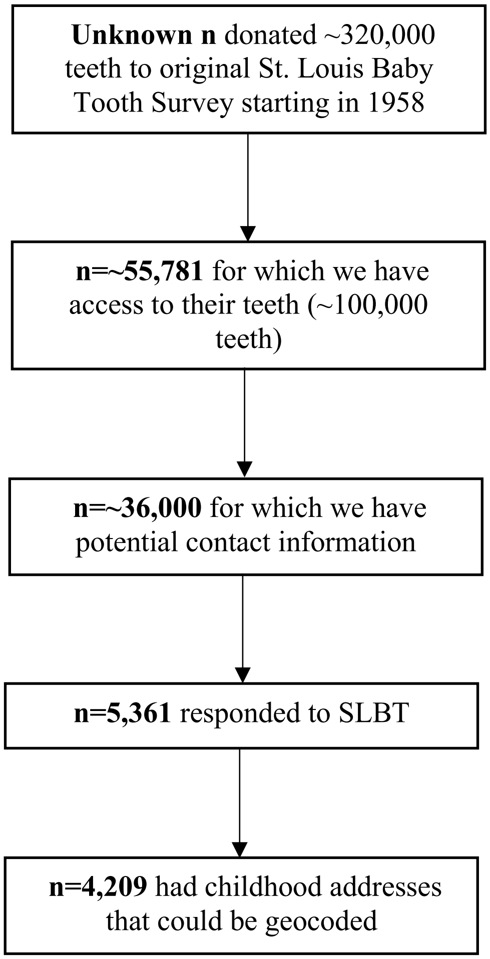


Participant selection in the St. Louis Baby Tooth Study Later Life Health Study (SLBT)

© 2025 Leung M et al. *JAMA Network Open*.

**eFigure 2.** Association Between Distance From Coldwater Creek and the Incidence of Any Form of Cancer

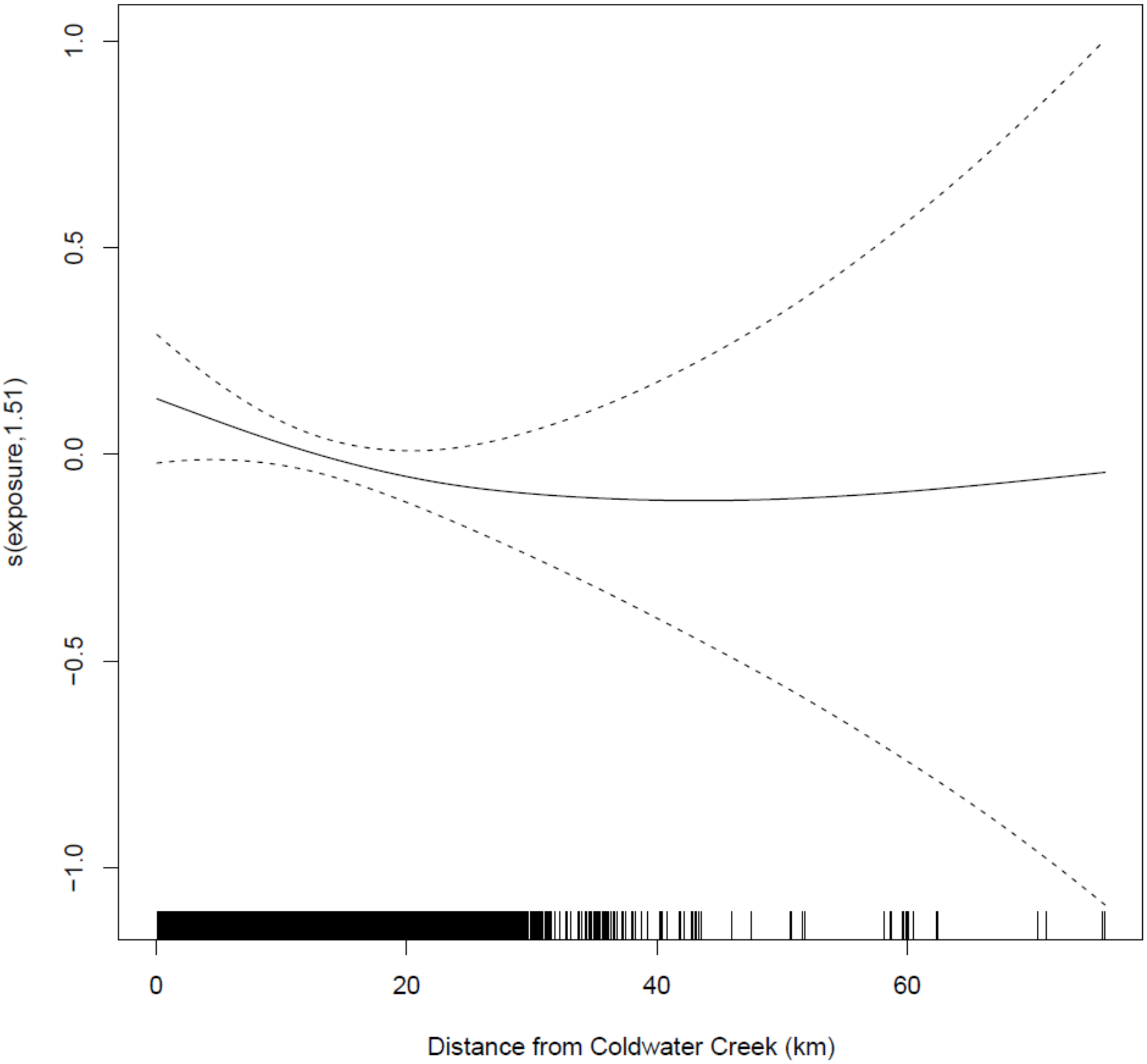


Association between distance from Coldwater Creek (kilometers, km) and the incidence of any form of cancer estimated using a generalized additive model fitted to participants from the St. Louis Baby Tooth—Later Life Health Study (n=4,209). The model was adjusted for sex, race/ethnicity, father's educational attainment, economic status at age 12, birth decade and median income from the 1960 Decennial census. The solid line represents the predicted log odds of any form of cancer given that all other covariates are at their respective means. The dashed line represents the 95% confidence intervals.

**eFigure 3.** Association Between Distance From Coldwater Creek and the Incidence of Solid Cancer

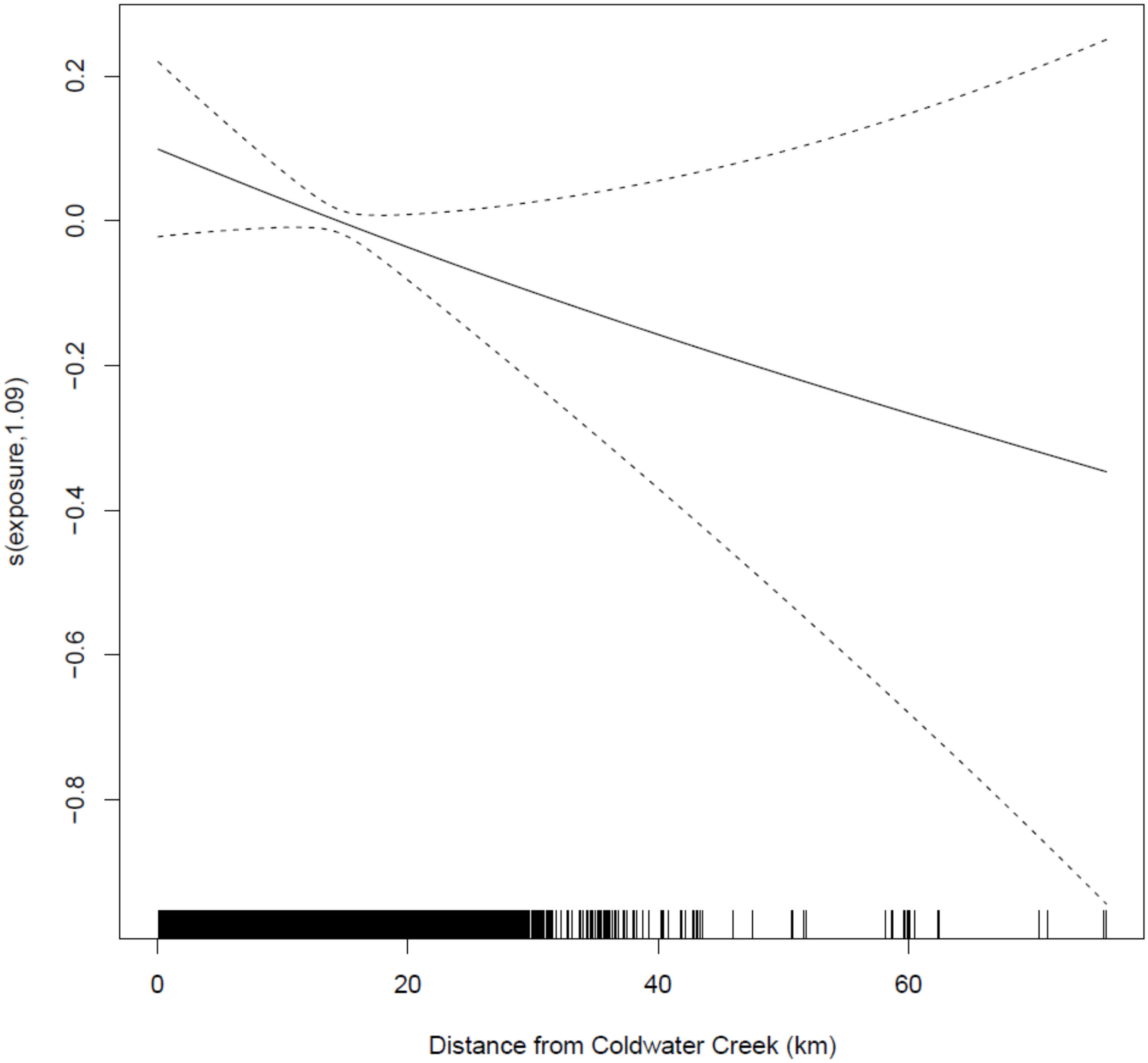


Association between distance from Coldwater Creek (kilometers, km) and the incidence of solid cancer estimated using a generalized additive model fitted to participants from the St. Louis Baby Tooth—Later Life Health Study (n=4,209). The model was adjusted for sex, race/ethnicity, father's educational attainment, economic status at age 12, birth decade and median income from the 1960 Decennial census. The solid line represents the predicted log odds of solid cancer given that all other covariates are at their respective means. The dashed line represents the 95% confidence intervals.

© 2025 Leung M et al. *JAMA Network Open*.

**eFigure 4.** Association Between Distance From Coldwater Creek and the Incidence of Radiosensitive Cancer

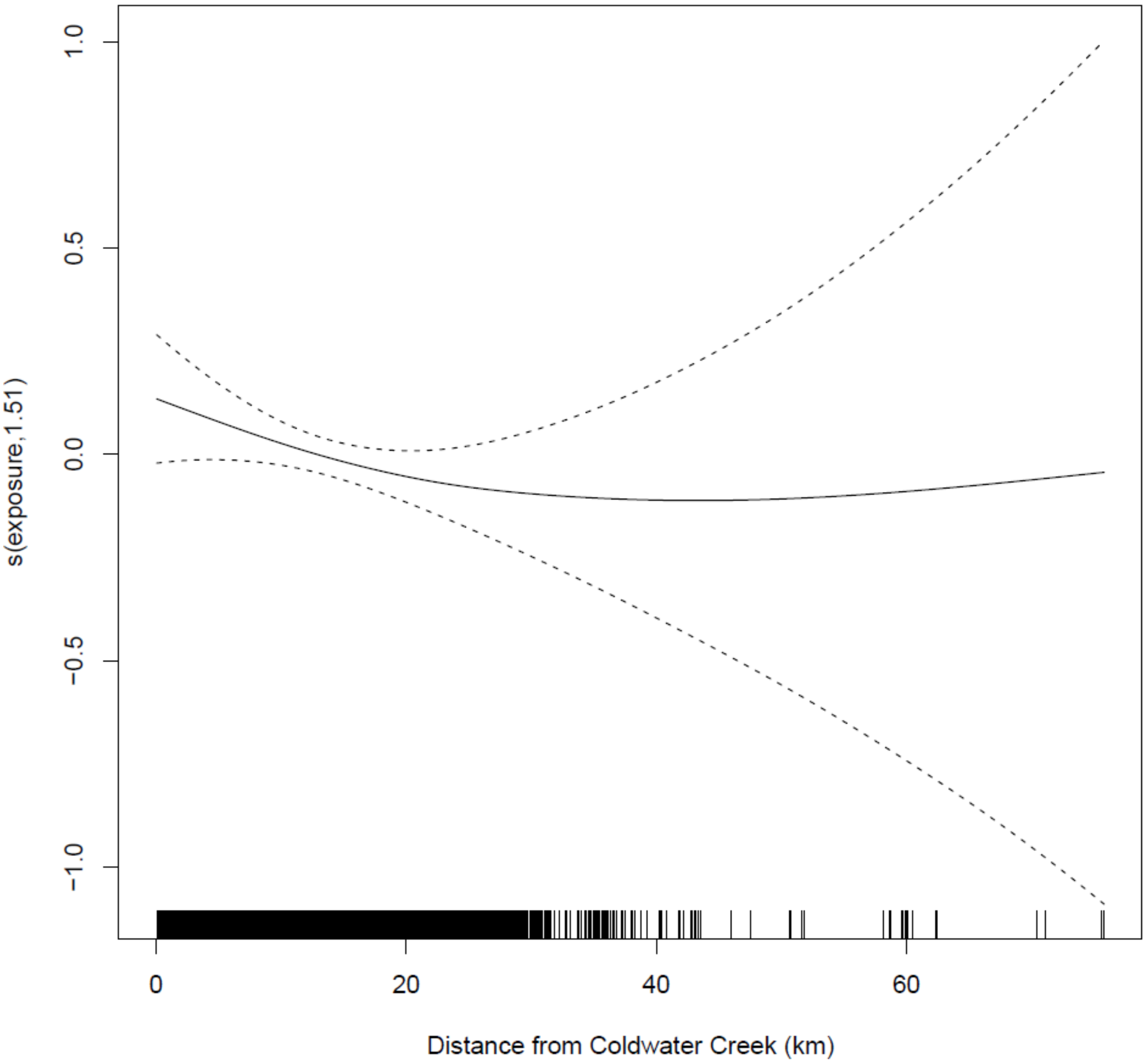


Association between distance from Coldwater Creek (kilometers, km) and the incidence of radiosensitive cancer estimated using a generalized additive model fitted to participants from the St. Louis Baby Tooth—Later Life Health Study (n=4,209). The model was adjusted for sex, race/ethnicity, father's educational attainment, economic status at age 12, birth decade and median income from the 1960 Decennial census. The solid line represents the predicted log odds of radiosensitive cancer given that all other covariates are at their respective means. The dashed line represents the 95% confidence intervals.

© 2025 Leung M et al. *JAMA Network Open*.

**eFigure 5.** Association Between Distance From Coldwater Creek and the Incidence of Nonradiosensitive Cancer

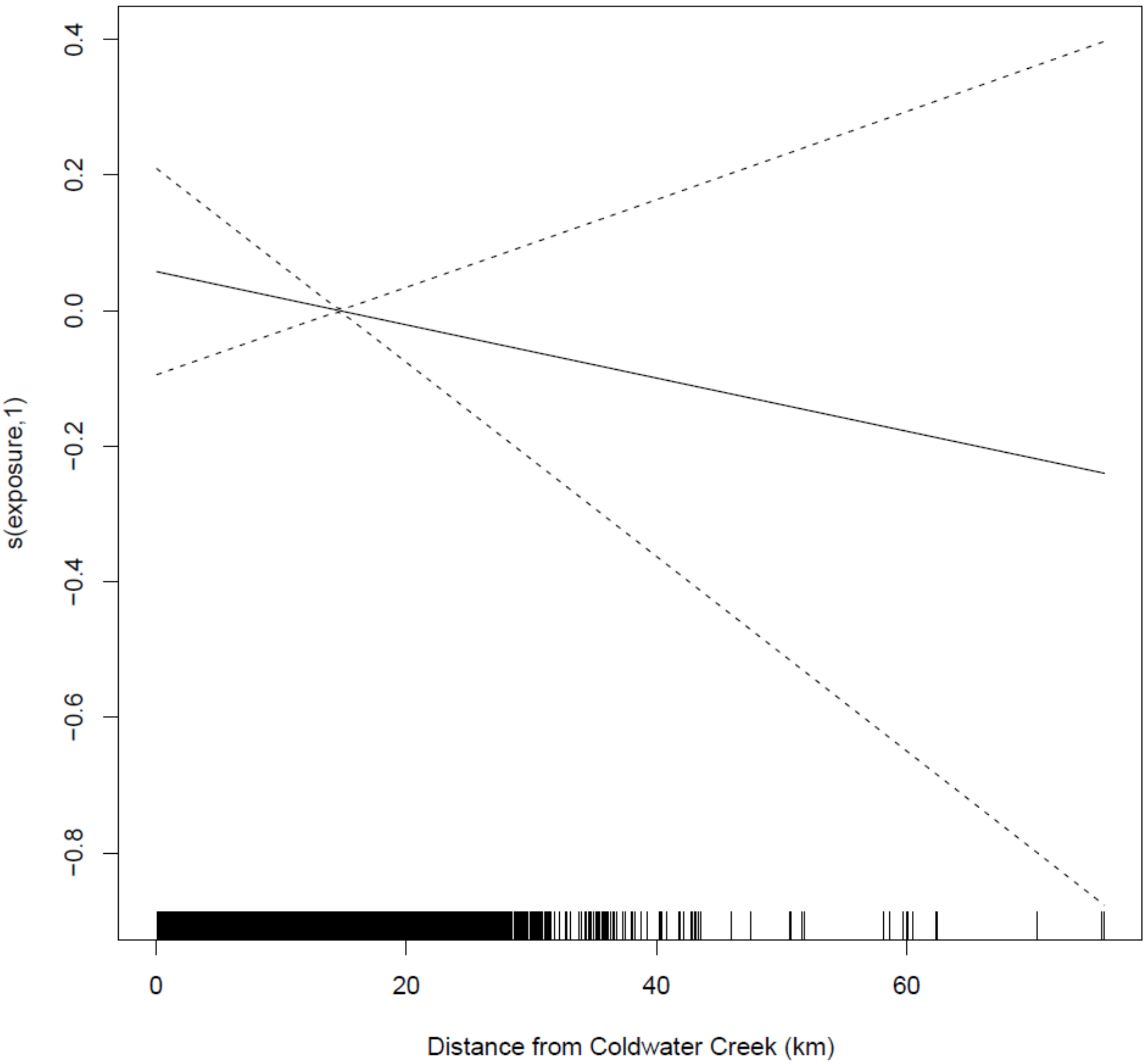


Association between distance from Coldwater Creek (kilometers, km) and the incidence of non-radiosensitive cancer estimated using a generalized additive model fitted to participants from the St. Louis Baby Tooth—Later Life Health Study (n=4,209). The model was adjusted for sex, race/ethnicity, father's educational attainment, economic status at age 12, birth decade and median income from the 1960 Decennial census. The solid line represents the predicted log odds of non-radiosensitive cancer given that all other covariates are at their respective means. The dashed line represents the 95% confidence intervals.

© 2025 Leung M et al. *JAMA Network Open*.

**eFigure 6.** Sex-Stratified Estimates for the Association Between Composite Cancer Outcomes and Proximity to Coldwater Creek

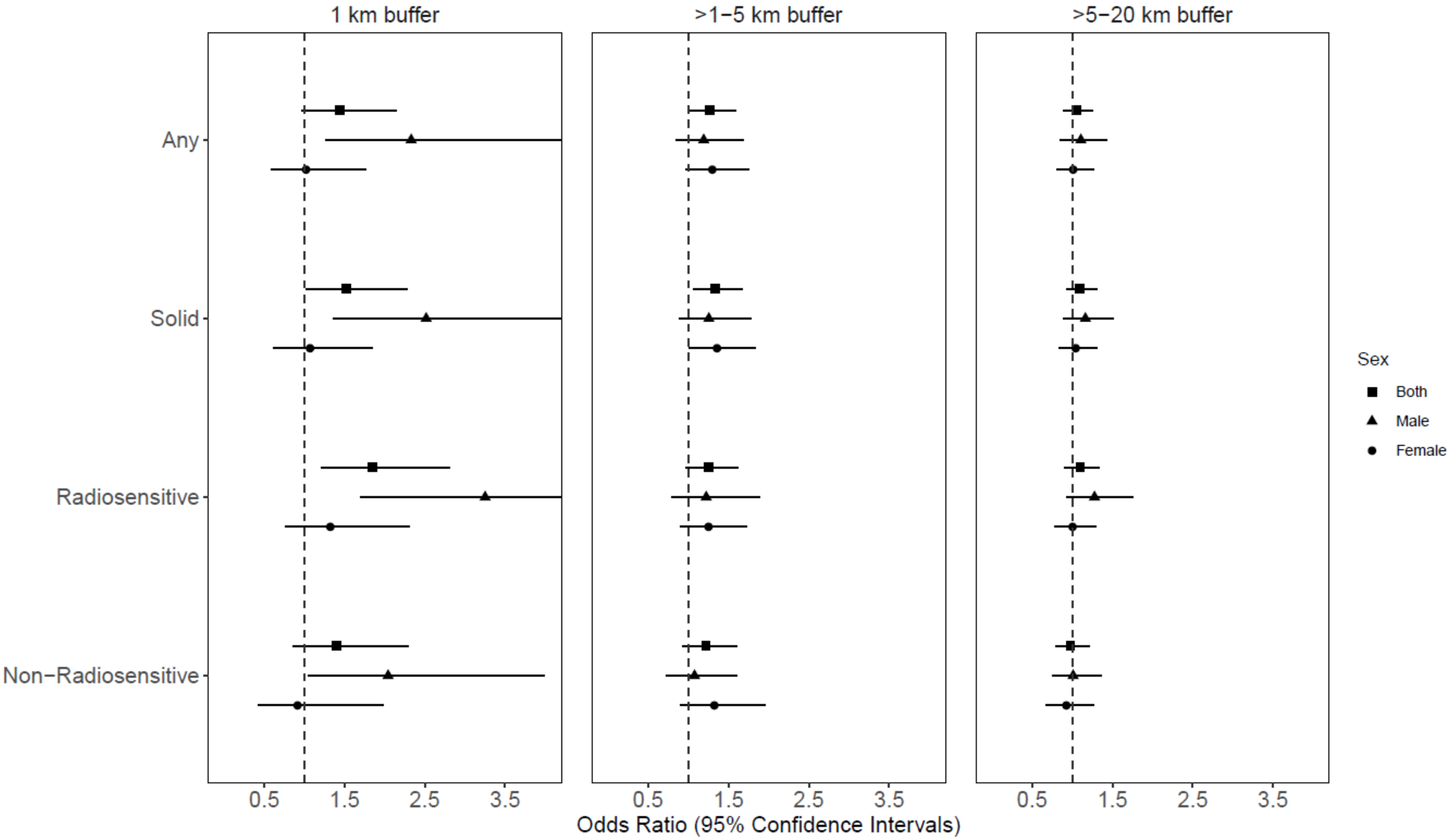


Sex-stratified estimates for the association between composite cancer outcomes (any, solid, radiosensitive, non-radiosensitive) and living ≤1 km, >1-5 km, and >5-20 km compared to living >20 km of Coldwater Creek, Missouri, 1945-1966 adjusted for race/ethnicity, father's educational attainment, economic status at age 12, birth decade and median income from the 1960 Decennial census. The point represents the odds ratio, and the error bars represent the 95% confidence interval (95% CI). The corresponding numeric data can be found in eTable 8.

© 2025 Leung M et al. *JAMA Network Open*.

**eFigure 7.** Sensitivity Analyses Estimates for the Association Between Composite Cancer Outcomes and Proximity to Coldwater Creek

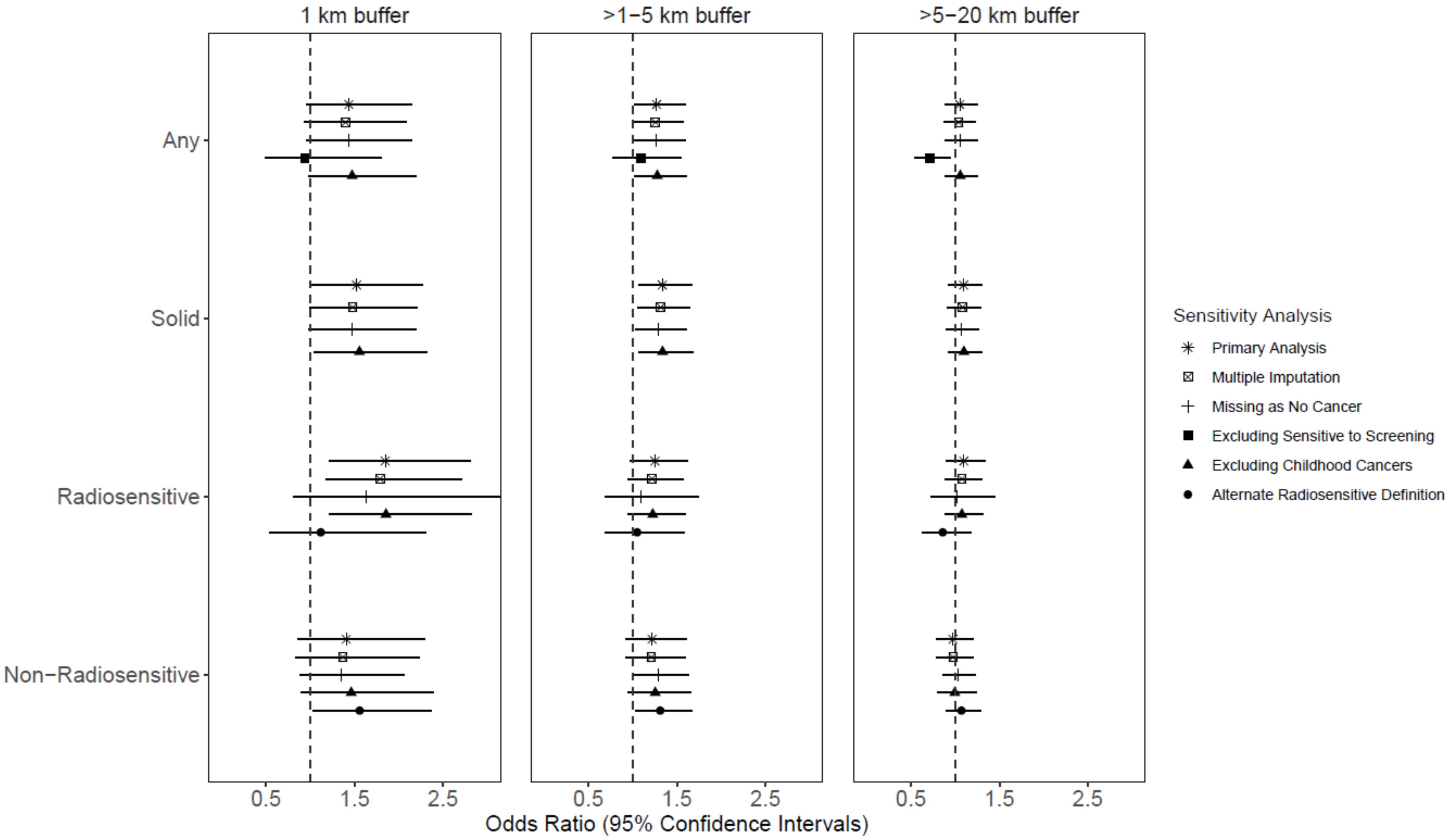


**eFigure 7.** Sensitivity analyses estimates for the association between composite cancer outcomes (any, solid, radiosensitive, non-radiosensitive) and living ≤1 km, >1-5 km, and >5-20 km compared to living >20 km of Coldwater Creek, Missouri, 1945-1966 adjusted for race/ethnicity,

© 2025 Leung M et al. *JAMA Network Open*.

father's educational attainment, economic status at age 12, birth decade and median income from the 1960 Decennial census. The point represents the odds ratio, and the error bars represent the 95% confidence interval (95% CI). The corresponding numeric data can be found in eTables 9-13.

© 2025 Leung M et al. *JAMA Network Open*.

**eFigure 8.** A Directed Acyclic Graph Representing the Bias That Could Arise From Conditioning on Cancer Survival and Study Participation

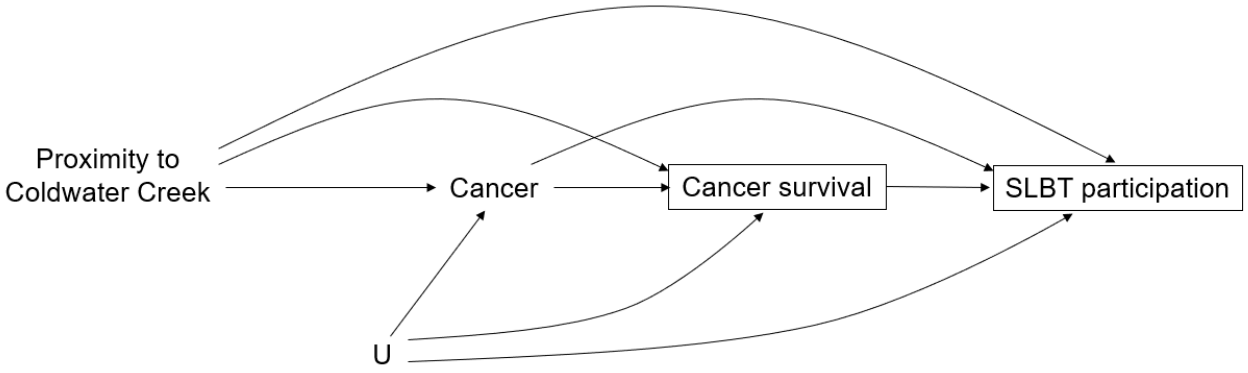


A directed acyclic graph representing the bias that could arise from conditioning on cancer survival and study participation. The *U*-variable represents potential unmeasured common causes of cancer incidence, cancer survival and study participation (e.g., underlying health status that may be unrelated to proximity to Coldwater Creek).

© 2025 Leung M et al. *JAMA Network Open*.

**eTable 1.** List of Counties in Greater St. Louis

| State | County |
|---|---|
| Illinois | Bond County |
| | Calhoun County |
| | Clinton County |
| | Jersey County |
| | Macoupin County |
| | Madison County |
| | Monroe County |
| | St. Clair County |
| Missouri | Franklin County |
| | Jefferson County |
| | Lincoln County |
| | St. Charles County |
| | St. Louis County |
| | Warren County |
| | City of St. Louis |

© 2025 Leung M et al. *JAMA Network Open*.

**eTable 2.** List of Cancers for Each Composite Outcome

| **Composite cancer outcome** | **Cancers included in grouping[a]** |
|---|---|
| Any | Breast, endometrial, cervical, ovarian, uterine, prostate, testicular, lung, colon, bladder, kidney, leukemia, liver, thyroid, non-Hodgkin's lymphoma, pancreas, melanoma, basal cell skin, squamous cell skin, Hodgkin's lymphoma |
| Solid | Breast, endometrial, cervical, ovarian, uterine, prostate, testicular, lung, colon, bladder, kidney, liver, thyroid, pancreas, melanoma, basal cell skin, squamous cell skin |
| Radiosensitive | Breast, thyroid, leukemia, basal cell skin |
| Non-radiosensitive | Endometrial, cervical, ovarian, uterine, prostate, testicular, lung, colon, bladder, kidney, liver, non-Hodgkin's lymphoma, pancreas, melanoma, squamous cell skin, Hodgkin's lymphoma |
| Radiosensitive, US Nuclear Regulatory Commission alternate definition | Breast, leukemia, bladder, colon, liver, lung, ovarian |
| Non-radiosensitive, alternate definition | Endometrial, cervical, uterine, prostate, testicular, kidney, thyroid, non-Hodgkin's lymphoma, pancreas, melanoma, basal cell skin, squamous cell skin, Hodgkin's lymphoma |
| Low sensitivity to screening intensity | Breast, endometrial, cervical, ovarian, uterine, testicular, lung, colon, bladder, kidney, leukemia, liver, non-Hodgkin's lymphoma, pancreas, Hodgkin's lymphoma |

[a] At enrollment, participants were asked if they were ever diagnosed with the following cancers: breast, endometrial, prostate, lung, colon, bladder, kidney, leukemia, liver, thyroid, non-Hodgkin's lymphoma, pancreatic, melanoma, basal cell, and squamous cell cancer. Participants also had the opportunity to provide a cancer outcome that was not listed above. We found that there were five or more cases of cervical, ovarian, uterine, testicular, and Hodgkin's lymphoma, and therefore we included these cancers in our analysis. If participants did not respond or report any cancer, we treated these data as missing.

© 2025 Leung M et al. *JAMA Network Open*.

**eTable 3.** Operationalization of Analytic Variables

| Variable | Analytic categories | Self-reported categories |
|---|---|---|
| Sex | 1: Male<br>0: Female | Male<br>Female |
| Race/Ethnicity | 1: Non-Hispanic White<br>0: Other | <u>Race</u><br>Black/African American<br>White<br>Asian<br>American Indian/Alaska Native<br>Native Hawaiian/Pacific Islander<br>Self-describe<br>Prefer not to say<br><br><u>Ethnicity</u><br>Hispanic or Latino<br>Not Hispanic or Latino |
| Dad education | 1: High school or less<br>2: Associate's degree or some college<br>3: College<br>4: More than college | Middle school or less (no high school)<br>Some high school<br>High school graduate<br>Some college<br>Associate degree (vocational/occupational/technical/academic program)<br>4-year college graduate<br>More than college |
| Economic status, age 12 | 1: Low income (Very poor, Poor)<br>2: Middle income (Average)<br>3: High income (Well off, Very well off) | Very poor<br>Poor<br>Average<br>Well off<br>Very well off |
| Birth decade | 1: 1940-1949<br>2: 1950-1959<br>3: ≥1960 | Birth year |

© 2025 Leung M et al. *JAMA Network Open*.

**eTable 4.** Site-Specific Cancer Status Through Ages 55 to 77 Stratified by Proximity to Coldwater Creek, Missouri, 1945 to 1966

| Cancer Type | | Residential Proximity to Coldwater Creek, n (%)[a] | | | |
|---|---|---|---|---|---|
| **Female Cancers** | | **≤1 km (n=83)** | **>1-5 km (n=348)** | **>5-20 km (n=1168)** | **>20 km (n=770)** |
| Breast | Non-case | 77 (92.8) | 323 (93.9) | 1066 (93.1) | 704 (93.2) |
| | Case | 6 (7.2) | 21 (6.1) | 79 (6.9) | 51 (6.8) |
| | Missing | 0 | 4 | 23 | 15 |
| Endometrial | Non-case | 81 (98.8) | 336 (97.4) | 1141 (99.4) | 745 (98.7) |
| | Case | 1 (1.2) | 9 (2.6) | 7 (0.6) | 10 (1.3) |
| | Missing | 1 | 3 | 20 | 15 |
| Cervical | Non-case | 83 (100.0) | 346 (99.4) | 1164 (99.7) | 766 (99.5) |
| | Case | 0 (0.0) | 2 (0.6) | 4 (0.3) | 4 (0.5) |
| | Missing | 0 | 0 | 0 | 0 |
| Ovarian | Non-case | 83 (100.0) | 345 (99.1) | 1160 (99.3) | 767 (99.6) |
| | Case | 0 (0.0) | 3 (0.9) | 8 (0.7) | 3 (0.4) |
| | Missing | 0 | 0 | 0 | 0 |
| Uterine | Non-case | 83 (100.0) | 348 (100.0) | 1163 (99.6) | 763 (99.1) |
| | Case | 0 (0.0) | 0 (0.0) | 5 (0.4) | 7 (0.9) |
| | Missing | 0 | 0 | 0 | 0 |
| **Male Cancers** | | **≤1 km (n=55)** | **>1-5 km (n=258)** | **>5-20 km (n=901)** | **>20 km (n=594)** |
| Prostate | Non-case | 47 (87.0) | 245 (96.1) | 833 (93.6) | 561 (95.9) |
| | Case | 7 (13.0) | 10 (3.9) | 57 (6.4) | 24 (4.1) |
| | Missing | 1 | 3 | 11 | 9 |
| Testicular | Non-case | 55 (100.0) | 254 (98.4) | 899 (99.8) | 593 (99.8) |
| | Case | 0 (0.0) | 4 (1.6) | 2 (0.2) | 1 (0.2) |
| | Missing | 0 | 0 | 0 | 0 |
| **Common Cancers** | | **≤1 km (n=139)** | **>1-5 km (n=609)** | **>5-20 km (n=2086)** | **>20 km (n=1375)** |
| Lung | Non-case | 137 (99.3) | 601 (99.7) | 2033 (99.7) | 1348 (99.8) |
| | Case | 1 (0.7) | 2 (0.3) | 7 (0.3) | 3 (0.2) |
| | Missing | 1 | 6 | 46 | 24 |
| Colon | Non-case | 137 (99.3) | 593 (98.5) | 2025 (99.4) | 1340 (99.3) |
| | Case | 1 (0.7) | 9 (1.5) | 13 (0.6) | 9 (0.7) |
| | Missing | 1 | 7 | 48 | 26 |
| Bladder | Non-case | 137 (99.3) | 602 (99.8) | 2039 (99.8) | 1346 (99.6) |
| | Case | 1 (0.7) | 1 (0.2) | 5 (0.2) | 6 (0.4) |
| | Missing | 1 | 6 | 42 | 23 |
| Kidney | Non-case | 138 (100.0) | 598 (99.5) | 2037 (99.7) | 1351 (99.7) |
| | Case | 0 (0.0) | 3 (0.5) | 6 (0.3) | 4 (0.3) |
| | Missing | 1 | 8 | 43 | 20 |
| Leukemia | Non-case | 137 (99.3) | 602 (99.8) | 2034 (99.9) | 1350 (99.6) |

© 2025 Leung M et al. *JAMA Network Open*.

| Cancer Type | | Residential Proximity to Coldwater Creek, n (%)[a] | | | |
|---|---|---|---|---|---|
| | Case | 1 (0.7) | 1 (0.2) | 3 (0.1) | 5 (0.4) |
| | Missing | 1 | 6 | 49 | 20 |
| Liver | Non-case | 138 (100.0) | 602 (100.0) | 2043 (100.0) | 1351 (99.9) |
| | Case | 0 (0.0) | 0 (0.0) | 0 (0.0) | 1 (0.1) |
| | Missing | 1 | 7 | 43 | 23 |
| Thyroid | Non-case | 135 (97.8) | 594 (98.7) | 2028 (99.3) | 1350 (99.6) |
| | Case | 3 (2.2) | 8 (1.3) | 15 (0.7) | 6 (0.4) |
| | Missing | 1 | 7 | 43 | 19 |
| Non-Hodgkin's Lymphoma | Non-case | 137 (99.3) | 600 (99.7) | 2030 (99.7) | 1346 (99.3) |
| | Case | 1 (0.7) | 2 (0.3) | 7 (0.3) | 10 (0.7) |
| | Missing | 1 | 7 | 49 | 19 |
| Pancreas | Non-case | 138 (100.0) | 600 (99.7) | 2041 (100.0) | 1351 (100.0) |
| | Case | 0 (0.0) | 2 (0.3) | 1 (0.0) | 0 (0.0) |
| | Missing | 1 | 7 | 44 | 24 |
| Melanoma | Non-case | 130 (94.2) | 578 (95.9) | 1968 (96.6) | 1302 (96.7) |
| | Case | 8 (5.8) | 25 (4.1) | 70 (3.4) | 44 (3.3) |
| | Missing | 1 | 6 | 48 | 29 |
| Basal cell skin | Non-case | 112 (81.2) | 517 (85.9) | 1776 (87.1) | 1189 (87.9) |
| | Case | 26 (18.8) | 85 (14.1) | 262 (12.9) | 163 (12.1) |
| | Missing | 1 | 7 | 48 | 23 |
| Squamous cell skin | Non-case | 126 (94.7) | 558 (93.9) | 1885 (93.2) | 1267 (94.0) |
| | Case | 7 (5.3) | 36 (6.1) | 137 (6.8) | 81 (6.0) |
| | Missing | 6 | 15 | 64 | 27 |
| Hodgkin's Lymphoma | Non-case | 139 (100.0) | 607 (99.7) | 2085 (100.0) | 1370 (99.6) |
| | Case | 0 (0.0) | 2 (0.3) | 1 (0.0) | 5 (0.4) |
| | Missing | 0 | 0 | 0 | 0 |

[a] Number of missing were not included in the calculation of %'s

© 2025 Leung M et al. *JAMA Network Open*.

**eTable 5.** Background Counterfactual Risks Obtained Through G-Computation for Several Cancer Outcomes

| Cancer Type | Background number of cases per 10,000 (95% CI) |
|---|---|
| **Composite Cancer Outcome** | |
| Any | 2412 (2177, 2659) |
| Solid | 2306 (2078, 2549) |
| Radiosensitive | 1542 (1367, 1746) |
| Non-Radiosensitive | 1383 (1202, 1590) |
| **Female Cancers** | |
| Breast | 683 (519, 854) |
| Endometrial | 134 (57, 222) |
| Cervical | 62 (12, 123) |
| Ovarian | 42 (0, 99) |
| Uterine | 93 (28, 167) |
| **Male Cancers** | |
| Prostate | 446 (291, 648) |
| Testicular | 20 (0, 209) |
| **Common Cancers** | |
| Lung | 23 (0, 52) |
| Colon | 68 (29, 121) |
| Bladder | 55 (16, 105) |
| Kidney | 33 (7, 74) |
| Leukemia | 34 (7, 67) |
| Liver | 6 (0, 22) |
| Thyroid | 45 (8, 90) |
| Non-Hodgkin's Lymphoma | 85 (38, 148) |
| Pancreas | 0 (0, 5) |
| Melanoma | 331 (238, 435) |
| Basal Cell Skin | 1207 (1021, 1394) |
| Squamous Cell Skin | 610 (478, 737) |
| Hodgkin's Lymphoma | 40 (10, 81) |

© 2025 Leung M et al. *JAMA Network Open*.

**eTable 6.** Estimates for the association between several cancer outcomes and Proximity to Coldwater Creek, Missouri, 1945 to 1966

| Cancer Type | ≤1 km | | >1-5 km | | >5-20 km | |
|---|---|---|---|---|---|---|
| | Case / Non-case | OR (95% CI)[a] | Case / Non-case | OR (95% CI)[a] | Case / Non-case | OR (95% CI)[a] |
| **Composite Cancer Outcome** | | | | | | |
| Any | 39 / 91 | 1.44 (0.96, 2.14) | 164 / 419 | 1.27 (1.01, 1.59) | 495 / 1461 | 1.05 (0.89, 1.25) |
| Solid | 39 / 91 | 1.52 (1.02, 2.28) | 163 / 421 | 1.33 (1.06, 1.67) | 492 / 1477 | 1.10 (0.92, 1.30) |
| Radiosensitive | 34 / 104 | 1.85 (1.21, 2.81) | 109 / 490 | 1.25 (0.97, 1.62) | 336 / 1680 | 1.10 (0.90, 1.34) |
| Non-Radiosensitive | 22 / 108 | 1.41 (0.86, 2.30) | 93 / 494 | 1.22 (0.92, 1.61) | 277 / 1703 | 0.98 (0.79, 1.21) |
| **Female Cancers** | | | | | | |
| Breast | 6 / 77 | 1.08 (0.45, 2.62) | 21 / 323 | 0.90 (0.53, 1.52) | 79 / 1066 | 0.99 (0.68, 1.45) |
| Endometrial | 1 / 81 | 0.86 (0.11, 6.87) | 9 / 336 | 2.12 (0.84, 5.35) | 7 / 1141 | 0.39 (0.14, 1.09) |
| Cervical | 0 / 83 | - | 2 / 346 | 0.96 (0.17, 5.32) | 4 / 1164 | 0.49 (0.10, 2.38) |
| Ovarian | 0 / 83 | - | 3 / 345 | 2.35 (0.47, 11.83) | 8 / 1160 | 1.40 (0.35, 5.61) |
| Uterine | 0 / 83 | - | 0 / 348 | - | 5 / 1163 | 0.47 (0.14, 1.51) |
| **Male Cancers** | | | | | | |
| Prostate | 7 / 47 | 3.96 (1.58, 9.94) | 10 / 245 | 0.76 (0.34, 1.67) | 57 / 833 | 1.44 (0.86, 2.39) |
| Testicular | 0 / 55 | - | 4 / 254 | 11.73 (1.27, 108.21) | 2 / 899 | 0.93 (0.07, 11.79) |
| **Common Cancers** | | | | | | |
| Lung | 1 / 137 | 3.33 (0.34, 32.94) | 2 / 601 | 1.65 (0.27, 9.98) | 7 / 2033 | 1.48 (0.37, 5.93) |
| Colon | 1 / 137 | 1.07 (0.13, 8.54) | 9 / 593 | 2.32 (0.91, 5.91) | 13 / 2025 | 0.75 (0.30, 1.89) |
| Bladder | 1 / 137 | 2.63 (0.30, 23.29) | 1 / 602 | 0.38 (0.04, 3.24) | 5 / 2039 | 0.29 (0.07, 1.19) |
| Kidney | 0 / 138 | - | 3 / 598 | 1.68 (0.37, 7.61) | 6 / 2037 | 0.87 (0.23, 3.26) |
| Leukemia | 1 / 137 | 2.31 (0.26, 20.77) | 1 / 602 | 0.54 (0.06, 4.84) | 3 / 2034 | 0.45 (0.10, 1.94) |
| Liver | 0 / 138 | - | 0 / 602 | - | 0 / 2043 | - |
| Thyroid | 3 / 135 | 5.00 (1.23, 20.32) | 8 / 594 | 3.13 (1.08, 9.10) | 15 / 2028 | 1.67 (0.64, 4.37) |
| Non-Hodgkin's Lymphoma | 1 / 137 | 1.02 (0.13, 8.13) | 2 / 600 | 0.43 (0.09, 1.98) | 7 / 2030 | 0.32 (0.11, 0.96) |
| Pancreas | 0 / 138 | - | 2 / 600 | - | 1 / 2041 | - |
| Melanoma | 8 / 130 | 1.97 (0.90, 4.31) | 25 / 578 | 1.35 (0.81, 2.24) | 70 / 1968 | 1.02 (0.68, 1.52) |
| Basal Cell Skin | 26 / 112 | 1.79 (1.13, 2.83) | 85 / 517 | 1.23 (0.93, 1.64) | 262 / 1776 | 1.07 (0.86, 1.33) |
| Squamous Cell Skin | 7 / 126 | 0.91 (0.41, 2.02) | 36 / 558 | 1.02 (0.67, 1.54) | 137 / 1885 | 1.11 (0.82, 1.49) |
| Hodgkin's Lymphoma | 0 / 139 | - | 2 / 607 | 0.85 (0.16, 4.39) | 1 / 2085 | 0.11 (0.01, 1.25) |

[a] Some cancers were rare, and so for several site-specific cancers, associations could not be estimated, but these individuals still contributed to the composite cancer outcomes.

© 2025 Leung M et al. *JAMA Network Open*.

**eTable 7.** Additional Cases per 10 000 for the Association Between Several Cancer Outcomes and Proximity to Coldwater Creek, Missouri, 1945 to 1966

| Cancer Type | ≤1 km | | >1-5 km | | >5-20 km | |
|---|---|---|---|---|---|---|
| | Case / Non-case | Cases/10,000 (95% CI)[a] | Case / Non-case | Cases/10,000 (95% CI)[a] | Case / Non-case | Cases/10,000 (95% CI)[a] |
| **Composite Cancer Outcome** | | | | | | |
| Any | 39 / 91 | 713 (8, 1598) | 164 / 419 | 460 (18, 888) | 495 / 1461 | 93 (-263, 415) |
| Solid | 39 / 91 | 817 (89, 1706) | 163 / 421 | 543 (131, 960) | 492 / 1477 | 164 (-188, 479) |
| Radiosensitive | 34 / 104 | 965 (277, 1883) | 109 / 490 | 313 (-29, 708) | 336 / 1680 | 125 (-157, 351) |
| Non-Radiosensitive | 22 / 108 | 446 (-174, 1120) | 93 / 494 | 247 (-126, 583) | 277 / 1703 | -29 (-273, 230) |
| **Cancers Among Women** | | | | | | |
| Breast | 6 / 77 | 53 (-491, 612) | 21 / 323 | -67 (-380, 248) | 79 / 1066 | -4 (-241, 235) |
| Endometrial | 1 / 81 | -18 (-209, 289) | 9 / 336 | 143 (-57, 327) | 7 / 1141 | -81 (-180, 4) |
| Cervical | 0 / 83 | - | 2 / 346 | -2 (-98, 113) | 4 / 1164 | -31 (-103, 31) |
| Ovarian | 0 / 83 | - | 3 / 345 | 55 (-52, 192) | 8 / 1160 | 16 (-50, 75) |
| Uterine | 0 / 83 | - | 0 / 348 | - | 5 / 1163 | -49 (-136, 23) |
| **Cancers Among Men** | | | | | | |
| Prostate | 7 / 47 | 1048 (106, 2079) | 10 / 245 | -102 (-377, 191) | 57 / 833 | 177 (-76, 430) |
| Testicular | 0 / 55 | - | 4 / 254 | 200 (10, 460) | 2 / 899 | -1 (-209, 43) |
| **Cancers Among Men and Women** | | | | | | |
| Lung | 1 / 137 | 53 (-46, 259) | 2 / 601 | 15 (-35, 86) | 7 / 2033 | 11 (-27, 51) |
| Colon | 1 / 137 | 5 (-114, 163) | 9 / 593 | 88 (-33, 194) | 13 / 2025 | -17 (-76, 34) |
| Bladder | 1 / 137 | 80 (-87, 351) | 1 / 602 | -33 (-93, 27) | 5 / 2039 | -38 (-89, 1) |
| Kidney | 0 / 138 | - | 3 / 598 | 22 (-43, 100) | 6 / 2037 | -4 (-50, 35) |
| Leukemia | 1 / 137 | 44 (-55, 272) | 1 / 602 | -16 (-58, 34) | 3 / 2034 | -19 (-54, 16) |
| Liver | 0 / 138 | - | 0 / 602 | - | 0 / 2043 | - |
| Thyroid | 3 / 135 | 176 (-37, 449) | 8 / 594 | 95 (-15, 204) | 15 / 2028 | 30 (-28, 90) |
| Non-Hodgkin's Lymphoma | 1 / 137 | 2 (-127, 239) | 2 / 600 | -48 (-121, 23) | 7 / 2030 | -57 (-126, -2) |
| Pancreas | 0 / 138 | - | 2 / 600 | - | 1 / 2041 | 4 (-5, 13) |
| Melanoma | 8 / 130 | 299 (-108, 708) | 25 / 578 | 110 (-91, 291) | 70 / 1968 | 5 (-129, 123) |
| Basal Cell Skin | 26 / 112 | 755 (92, 1386) | 85 / 517 | 238 (-110, 558) | 262 / 1776 | 71 (-154, 309) |
| Squamous Cell Skin | 7 / 126 | -52 (-423, 369) | 36 / 558 | 11 (-197, 253) | 137 / 1885 | 60 (-96, 240) |
| Hodgkin's Lymphoma | 0 / 139 | - | 2 / 607 | -6 (-64, 57) | 1 / 2085 | -36 (-72, -5) |

[a] Some cancers were rare, and so for several site-specific cancers, associations could not be estimated, but these individuals still contributed to the composite cancer outcomes.

© 2025 Leung M et al. *JAMA Network Open*.

**eTable 8.** Sex-Stratified Estimates for the Association Between Several Cancer Outcomes and Proximity to Coldwater Creek, Missouri, 1945 to 1966

| Cancer Type | ≤1 km | | >1-5 km | | >5-20 km | |
|---|---|---|---|---|---|---|
| | Case / Non-case | OR (95% CI) | Case / Non-case | OR (95% CI) | Case / Non-case | OR (95% CI) |
| **Composite Cancer Outcome** | | | | | | |
| Any | 39 / 91 | 1.44 (0.96, 2.14) | 164 / 419 | 1.27 (1.01, 1.59) | 495 / 1461 | 1.05 (0.89, 1.25) |
| Solid | 39 / 91 | 1.52 (1.02, 2.28) | 163 / 421 | 1.33 (1.06, 1.67) | 492 / 1477 | 1.10 (0.92, 1.30) |
| Radiosensitive | 34 / 104 | 1.85 (1.21, 2.81) | 109 / 490 | 1.25 (0.97, 1.62) | 336 / 1680 | 1.10 (0.90, 1.34) |
| Non-Radiosensitive | 22 / 108 | 1.41 (0.86, 2.30) | 93 / 494 | 1.22 (0.92, 1.61) | 277 / 1703 | 0.98 (0.79, 1.21) |
| **Male** | | | | | | |
| Any | 20 / 30 | 2.33 (1.27, 4.29) | 69 / 180 | 1.19 (0.84, 1.69) | 225 / 630 | 1.11 (0.85, 1.43) |
| Solid | 20 / 30 | 2.52 (1.37, 4.64) | 68 / 181 | 1.26 (0.88, 1.78) | 222 / 637 | 1.16 (0.89, 1.51) |
| Radiosensitive | 16 / 38 | 3.25 (1.70, 6.20) | 37 / 218 | 1.22 (0.79, 1.88) | 133 / 746 | 1.28 (0.93, 1.76) |
| Non-Radiosensitive | 14 / 36 | 2.04 (1.05, 3.99) | 47 / 202 | 1.08 (0.72, 1.61) | 155 / 706 | 1.01 (0.75, 1.36) |
| **Female** | | | | | | |
| Any | 19 / 60 | 1.02 (0.59, 1.77) | 95 / 236 | 1.30 (0.96, 1.75) | 269 / 820 | 1.01 (0.80, 1.27) |
| Solid | 19 / 60 | 1.07 (0.62, 1.85) | 95 / 237 | 1.36 (1.01, 1.83) | 268 / 829 | 1.04 (0.83, 1.31) |
| Radiosensitive | 18 / 65 | 1.32 (0.76, 2.31) | 72 / 269 | 1.25 (0.90, 1.73) | 202 / 923 | 1.00 (0.78, 1.29) |
| Non-Radiosensitive | 8 / 71 | 0.91 (0.42, 1.98) | 46 / 289 | 1.32 (0.89, 1.96) | 121 / 985 | 0.93 (0.68, 1.27) |

© 2025 Leung M et al. *JAMA Network Open*.

**eTable 9.** Estimates for the Association Between Several Cancer Outcomes and Proximity to Coldwater Creek, Missouri, 1945 to 1966, After Multiple Imputation

| Cancer Type | ≤1 km | | >1-5 km | | >5-20 km | |
|---|---|---|---|---|---|---|
| | Case / Non-case | OR (95% CI)[a] | Case / Non-case | OR (95% CI)[a] | Case / Non-case | OR (95% CI)[a] |
| **Composite Cancer Outcome** | | | | | | |
| Any | 39 / 91 | 1.40 (0.94, 2.08) | 164 / 419 | 1.26 (1.01, 1.57) | 495 / 1461 | 1.04 (0.88, 1.23) |
| Solid | 39 / 91 | 1.48 (0.99, 2.21) | 163 / 421 | 1.32 (1.05, 1.65) | 492 / 1477 | 1.08 (0.91, 1.29) |
| Radiosensitive | 34 / 104 | 1.79 (1.18, 2.72) | 109 / 490 | 1.22 (0.94, 1.57) | 336 / 1680 | 1.08 (0.89, 1.31) |
| Non-Radiosensitive | 22 / 108 | 1.37 (0.84, 2.23) | 93 / 494 | 1.21 (0.92, 1.59) | 277 / 1703 | 0.98 (0.79, 1.21) |

[a] We used multiple imputation to create 10 completed datasets from which estimates were pooled by applying Rubin's rules

© 2025 Leung M et al. *JAMA Network Open*.

**eTable 10.** Estimates for the Association Between Several Cancer Outcomes and Proximity to Coldwater Creek, Missouri, 1945 to 1966, After Treating Missing Cancer Outcomes as No Cancer

| Cancer Type | ≤1 km | | >1-5 km | | >5-20 km | |
|---|---|---|---|---|---|---|
| | Case / Non-case | OR (95% CI) | Case / Non-case | OR (95% CI) | Case / Non-case | OR (95% CI) |
| **Composite Cancer Outcome** | | | | | | |
| Any | 39 / 100 | 1.44 (0.96, 2.14) | 164 / 445 | 1.27 (1.01, 1.59) | 495 / 1591 | 1.05 (0.89, 1.25) |
| Solid | 39 / 100 | 1.47 (0.98, 2.19) | 163 / 446 | 1.29 (1.03, 1.61) | 492 / 1594 | 1.06 (0.90, 1.26) |
| Radiosensitive | 34 / 105 | 1.63 (0.80, 3.32) | 109 / 500 | 1.10 (0.69, 1.75) | 336 / 1750 | 1.02 (0.72, 1.46) |
| Non-Radiosensitive | 22 / 117 | 1.35 (0.88, 2.07) | 93 / 516 | 1.29 (1.02, 1.64) | 277 / 1809 | 1.03 (0.86, 1.24) |

© 2025 Leung M et al. *JAMA Network Open*.

**eTable 11.** Estimates for the Association Between Several Cancer Outcomes and Proximity to Coldwater Creek, Missouri, 1945 to 1966, After Excluding Individuals With Childhood Cancers

| Cancer Type | ≤1 km | | >1-5 km | | >5-20 km | |
|---|---|---|---|---|---|---|
| | Case / Non-case | OR (95% CI)[a] | Case / Non-case | OR (95% CI)[a] | Case / Non-case | OR (95% CI)[a] |
| **Composite Cancer Outcome** | | | | | | |
| Any | 39 / 91 | 1.47 (0.99, 2.20) | 161 / 419 | 1.28 (1.02, 1.60) | 486 / 1461 | 1.06 (0.89, 1.26) |
| Solid | 39 / 91 | 1.56 (1.04, 2.33) | 160 / 421 | 1.34 (1.06, 1.68) | 483 / 1477 | 1.10 (0.92, 1.31) |
| Radiosensitive | 34 / 104 | 1.86 (1.22, 2.82) | 106 / 489 | 1.23 (0.94, 1.59) | 328 / 1678 | 1.08 (0.88, 1.31) |
| Non-Radiosensitive | 22 / 108 | 1.46 (0.89, 2.40) | 92 / 492 | 1.25 (0.95, 1.66) | 272 / 1699 | 1.00 (0.80, 1.24) |

[a] Associations with childhood cancers could not be examined as there were only 23 cases in the study.

© 2025 Leung M et al. *JAMA Network Open*.

**eTable 12.** Estimates for the Association Between Any Cancer and Proximity to Coldwater Creek, Missouri, 1945 to 1966, After Restricting to Cancers That Are Not Sensitive to Screening Intensity

| Cancer Type | ≤1 km | | >1-5 km | | >5-20 km | |
|---|---|---|---|---|---|---|
| | Case / Non-case | OR (95% CI) | Case / Non-case | OR (95% CI) | Case / Non-case | OR (95% CI) |
| Any Cancer[a] | 39 / 91 | 1.44 (0.96, 2.14) | 164 / 419 | 1.27 (1.01, 1.59) | 495 / 1461 | 1.05 (0.89, 1.25) |
| Cancers Non-Sensitive to Screening[b] | 11 / 126 | 0.94 (0.49, 1.80) | 54 / 541 | 1.09 (0.77, 1.55) | 137 / 1866 | 0.71 (0.54, 0.94) |

[a] Breast, endometrial, cervical, ovarian, uterine, prostate, testicular, lung, colon, bladder, kidney, leukemia, liver, thyroid, non-Hodgkin's lymphoma, pancreas, melanoma, basal cell skin, squamous cell skin, Hodgkin's lymphoma

[b] Breast, endometrial, cervical, ovarian, uterine, testicular, lung, colon, bladder, kidney, leukemia, liver, non-Hodgkin's lymphoma, pancreas, Hodgkin's lymphoma

© 2025 Leung M et al. *JAMA Network Open*.

**eTable 13.** Estimates for the Association Between Radiosensitive Cancer and Proximity to Coldwater Creek, Missouri, 1945 to 1966, After Using an Alternate Definition of "Radiosensitive" Based on Cancers Identified by the US Nuclear Regulatory Commission

| Any Cancer | ≤1 km | | >1-5 km | | >5-20 km | |
|---|---|---|---|---|---|---|
| | Case / Non-case | OR (95% CI) | Case / Non-case | OR (95% CI) | Case / Non-case | OR (95% CI) |
| **Primary Analysis** | | | | | | |
| Radiosensitive | 34 / 104 | 1.85 (1.21, 2.81) | 109 / 490 | 1.25 (0.97, 1.62) | 336 / 1680 | 1.10 (0.90, 1.34) |
| Non-Radiosensitive | 22 / 108 | 1.41 (0.86, 2.30) | 93 / 494 | 1.22 (0.92, 1.61) | 277 / 1703 | 0.98 (0.79, 1.21) |
| **Alternate Definition of "Radiosensitive"** | | | | | | |
| Radiosensitive[a] | 9 / 129 | 1.12 (0.54, 2.31) | 36 / 564 | 1.05 (0.69, 1.59) | 109 / 1907 | 0.86 (0.63, 1.18) |
| Non-Radiosensitive[b] | 34 / 96 | 1.56 (1.02, 2.37) | 140 / 446 | 1.31 (1.03, 1.66) | 416 / 1565 | 1.07 (0.89, 1.29) |

[a] Radiosensitive: leukemia, bladder, breast, lung, ovarian, colon, liver

[b] Non-Radiosensitive: endometrial, cervical, uterine, prostate, testicular, kidney, thyroid, Non-Hodgkin's lymphoma, pancreas, melanoma, Hodgkin's lymphoma

© 2025 Leung M et al. *JAMA Network Open*.

**eTable 14.** E Values for the Estimates From the Primary Analysis to Assess for the Magnitude of Confounding That Would Be Needed to Explain Away the Point Estimate

| Cancer Type | ≤1 km[a] | | >1-5 km[a] | | >5-20 km[a] | |
|---|---|---|---|---|---|---|
| | OR (95% CI) | E-value | OR (95% CI) | E-value | OR (95% CI) | E-value |
| **Composite Cancer Outcome** | | | | | | |
| Any | 1.44 (0.96, 2.14) | 2.23 | 1.27 (1.01, 1.59) | 1.86 | 1.05 (0.89, 1.25) | 1.29 |
| Solid | 1.52 (1.02, 2.28) | 2.42 | 1.33 (1.06, 1.67) | 2 | 1.10 (0.92, 1.30) | 1.42 |
| Radiosensitive | 1.85 (1.21, 2.81) | 3.1 | 1.25 (0.97, 1.62) | 1.81 | 1.10 (0.90, 1.34) | 1.43 |
| Non-Radiosensitive | 1.41 (0.86, 2.30) | 2.16 | 1.22 (0.92, 1.61) | 1.74 | 0.98 (0.79, 1.21) | 1.19 |
| **Cancers Among Women** | | | | | | |
| Breast | 1.08 (0.45, 2.62) | 1.39 | 0.90 (0.53, 1.52) | 1.48 | 0.99 (0.68, 1.45) | 1.08 |
| Endometrial | 0.86 (0.11, 6.87) | 1.6 | 2.12 (0.84, 5.35) | 3.66 | 0.39 (0.14, 1.09) | 4.58 |
| Cervical | - | - | 0.96 (0.17, 5.32) | 1.24 | 0.49 (0.10, 2.38) | 3.46 |
| Ovarian | - | - | 2.35 (0.47, 11.83) | 4.13 | 1.40 (0.35, 5.61) | 2.14 |
| Uterine | - | - | - | - | 0.47 (0.14, 1.51) | 3.71 |
| **Cancers Among Men** | | | | | | |
| Prostate | 3.96 (1.58, 9.94) | 7.39 | 0.76 (0.34, 1.67) | 1.97 | 1.44 (0.86, 2.39) | 2.23 |
| Testicular | - | - | 11.73 (1.27, 108.21) | 22.96 | 0.93 (0.07, 11.79) | 1.35 |
| **Common Cancers** | | | | | | |
| Lung | 3.33 (0.34, 32.94) | 6.12 | 1.65 (0.27, 9.98) | 2.68 | 1.48 (0.37, 5.93) | 2.32 |
| Colon | 1.07 (0.13, 8.54) | 1.34 | 2.32 (0.91, 5.91) | 4.07 | 0.75 (0.30, 1.89) | 2 |
| Bladder | 2.63 (0.30, 23.29) | 4.7 | 0.38 (0.04, 3.24) | 4.73 | 0.29 (0.07, 1.19) | 6.25 |
| Kidney | - | - | 1.68 (0.37, 7.61) | 2.76 | 0.87 (0.23, 3.26) | 1.56 |
| Leukemia | 2.31 (0.26, 20.77) | 4.05 | 0.54 (0.06, 4.84) | 3.09 | 0.45 (0.10, 1.94) | 3.87 |
| Liver | - | - | - | - | - | - |
| Thyroid | 5.00 (1.23, 20.32) | 9.47 | 3.13 (1.08, 9.10) | 5.71 | 1.67 (0.64, 4.37) | 2.72 |
| Non-Hodgkin's Lymphoma | 1.02 (0.13, 8.13) | 1.18 | 0.43 (0.09, 1.98) | 4.09 | 0.32 (0.11, 0.96) | 5.67 |
| Pancreas | - | 1.68 | - | - | - | - |
| Melanoma | 1.97 (0.90, 4.31) | 3.36 | 1.35 (0.81, 2.24) | 2.04 | 1.02 (0.68, 1.52) | 1.15 |
| Basal Cell Skin | 1.79 (1.13, 2.83) | 2.97 | 1.23 (0.93, 1.64) | 1.77 | 1.07 (0.86, 1.33) | 1.34 |
| Squamous Cell Skin | 0.91 (0.41, 2.02) | 1.43 | 1.02 (0.67, 1.54) | 1.16 | 1.11 (0.82, 1.49) | 1.45 |
| Hodgkin's Lymphoma | - | - | 0.85 (0.16, 4.39) | 1.65 | 0.11 (0.01, 1.25) | 17.2 |

[a] Some cancers were rare, and so for several site-specific cancers, associations could not be estimated, but these individuals still contributed to the composite cancer outcomes.

© 2025 Leung M et al. *JAMA Network Open*.

**eTable 15.** Estimates for the Association Between SLBT Participation and Proximity to Coldwater Creek, Missouri, 1945 to 1966

| Adjustment | Odds ratio (95% CI) | | |
|---|---|---|---|
| | ≤1-km | >1-5-km | >5-20 km |
| None | 0.91 (0.76, 1.10) | 0.89 (0.80, 0.98) | 0.93 (0.87, 1.00) |
| Census tract-level median income + % Black | 0.89 (0.73, 1.05) | 0.85 (0.77, 0.94) | 0.93 (0.87, 1.00) |

© 2025 Leung M et al. *JAMA Network Open*.

**eTable 16**. Comparison of Our Additive Estimates (Excess Cases per 10 000) from the St. Louis Baby Tooth–Later Life Health Study (SLBT) With Those From the Agency for Toxic Substances and Disease Registry (ATSDR) Report

| Cancer Type | Additional cases per 10,000 (95% CI) | | | |
|---|---|---|---|---|
| | SLBT[a] | | | ATSDR[b] |
| | ≤1 km | >1-5 km | >5-20 km | |
| **Composite Cancer Outcome** | | | | |
| Any | 713 (8, 1598) | 460 (18, 888) | 93 (-263, 415) | - |
| Solid | 817 (89, 1706) | 543 (131, 960) | 164 (-188, 479) | - |
| Radiosensitive | 965 (277, 1883) | 313 (-29, 708) | 125 (-157, 351) | - |
| Non-Radiosensitive | 446 (-174, 1120) | 247 (-126, 583) | -29 (-273, 230) | - |
| **Cancers Among Women** | | | | |
| Breast | 53 (-491, 612) | -67 (-380, 248) | -4 (-241, 235) | 0.6 to 0.2 |
| Endometrial | -18 (-209, 289) | 143 (-57, 327) | -81 (-180, 4) | - |
| Cervical | - | -2 (-98, 113) | -31 (-103, 31) | - |
| Ovarian | - | 55 (-52, 192) | 16 (-50, 75) | 0.03 to 0.2 |
| Uterine | - | - | -49 (-136, 23) | 0.009 to 0.03 |
| **Cancers Among Men** | | | | |
| Prostate | 1048 (106, 2079) | -102 (-377, 191) | 177 (-76, 430) | - |
| Testicular | - | 200 (10, 460) | -1 (-209, 43) | - |
| **Common Cancers** | | | | |
| Lung | 53 (-46, 259) | 15 (-35, 86) | 11 (-27, 51) | 4 to 10 |
| Colon | 5 (-114, 163) | 88 (-33, 194) | -17 (-76, 34) | 0.1 to 0.4 |
| Bladder | 80 (-87, 351) | -33 (-93, 27) | -38 (-89, 1) | 0.06 to 0.1 |
| Kidney | - | 22 (-43, 100) | -4 (-50, 35) | 0.1 to 0.7 |
| Leukemia | 44 (-55, 272) | -16 (-58, 34) | -19 (-54, 16) | 1 to 4 |
| Liver | - | - | - | 0.08 to 0.6 |
| Thyroid | 176 (-37, 449) | 95 (-15, 204) | 30 (-28, 90) | 0.06 to 0.2 |
| Non-Hodgkin's Lymphoma | 2 (-127, 239) | -48 (-121, 23) | -57 (-126, -2) | - |
| Pancreas | - | - | 4 (-5, 13) | - |
| Melanoma | 299 (-108, 708) | 110 (-91, 291) | 5 (-129, 123) | 0.3 to 1 |
| Basal Cell Skin | 755 (92, 1386) | 238 (-110, 558) | 71 (-154, 309) | |
| Squamous Cell Skin | -52 (-423, 369) | 11 (-197, 253) | 60 (-96, 240) | |
| Hodgkin's Lymphoma | - | -6 (-64, 57) | -36 (-72, -5) | - |

[a] Some cancers were rare, and so for several site-specific cancers, associations could not be estimated, but these individuals still contributed to the composite cancer outcomes.
[b] Range represents the lifetime attributable risk depending on whether exposure was recreational versus residential and whether lung solubility was assumed to be slow versus medium. Confidence intervals were not reported, and there were no estimates for some sites either because they were not presented or there were no organ-specific attributable risk coefficients available.
