# Supplementary material for: Cancer Incidence and Childhood Residence Near the Coldwater Creek Radioactive Waste Site
Source: JAMA Netw Open. 2025 Jul 16;8(7):e2521926. doi: 10.1001/jamanetworkopen.2025.21926 (PMC12268482; doi:10.1001/jamanetworkopen.2025.21926)
Supplement: Supplement 2. — Data Sharing Statement [file jamanetwopen-e2521926-s002.pdf]

Data Sharing Statement

Leung. Cancer Incidence and Childhood Residence Near the Coldwater Creek Radioactive Waste Site. *JAMA Netw Open*. Published July 16, 2025.
doi:10.1001/jamanetworkopen.2025.21926

Data
Data available: No
